# Robust retrospective motion correction of head motion using navigator‐based and markerless motion tracking techniques

**DOI:** 10.1002/mrm.29705

**Published:** 2023-05-15

**Authors:** Elisa Marchetto, Kevin Murphy, Stefan L. Glimberg, Daniel Gallichan

**Affiliations:** ^1^ CUBRIC/School of Engineering Cardiff University Cardiff UK; ^2^ CUBRIC/School of Physics and Astronomy Cardiff University Cardiff UK; ^3^ TracInnovations Ballerup Denmark

**Keywords:** brain MRI, fat navigator, markerless head motion tracking, MRI motion artifact correction, retrospective motion correction

## Abstract

**Purpose:**

This study investigated the artifacts arising from different types of head motion in brain MR images and how well these artifacts can be compensated using retrospective correction based on two different motion‐tracking techniques.

**Methods:**

MPRAGE images were acquired using a 3 T MR scanner on a cohort of nine healthy participants. Subjects moved their head to generate circular motion (4 or 6 cycles/min), stepwise motion (small and large) and “simulated realistic” motion (nodding and slow diagonal motion), based on visual instructions. One MPRAGE scan without deliberate motion was always acquired as a “no motion” reference. Three dimensional fat‐navigator (FatNavs) and a Tracoline markerless device (TracInnovations) were used to obtain motion estimates and images were separately reconstructed retrospectively from the raw data based on these different motion estimates.

**Results:**

Image quality was recovered from both motion tracking techniques in our stepwise and slow diagonal motion scenarios in almost all cases, with the apparent visual image quality comparable to the no‐motion case. FatNav‐based motion correction was further improved in the case of stepwise motion using a skull masking procedure to exclude non‐rigid motion of the neck from the co‐registration step. In the case of circular motion, both methods struggled to correct for all motion artifacts.

**Conclusion:**

High image quality could be recovered in cases of stepwise and slow diagonal motion using both motion estimation techniques. The circular motion scenario led to more severe image artifacts that could not be fully compensated by the retrospective motion correction techniques used.

## 1 INTRODUCTION

Motion artifacts are a well‐known issue in MR images,^1^ which might impede the interpretation of a patient's condition and obscure pathologies and regions of interest. To address these problems, MRI acquisitions can be repeated, but this leads to discomfort for the patient and increased costs for clinical or research centres.^2^, ^3^ Moreover, clinical facilities often need to use anesthesia or sedation on children to reduce motion corruption of images, leading to an increased risk of acute adverse events^4^, ^5^ and higher financial costs.^3^, ^6^

Methods have been developed in MRI to estimate the motion that occurred and restore sharpness and resolution to reduce the need for reacquisition, which can be divided into two main categories^7^:

1. Retrospective motion correction—where the data collected during the scan is corrected for motion in post‐processing.
2. Prospective motion correction—consisting of real‐time correction by updating gradients and RF pulses during the acquisition.

Retrospective motion correction can often be simpler to implement from a practical perspective as it avoids the technical complications of real‐time feedback and has the additional advantage that the uncorrected image is also preserved, avoiding the potential for loss of image quality in the case of inaccuracies in motion‐tracking. By contrast, prospective techniques can be applied to a wider variety of pulse sequences and are generally more robust against motion artifacts, especially as they also enable the possibility of the immediate reacquisition of the most corrupted regions of k‐space.^8^

Both approaches can rely on different strategies to estimate the motion parameters such as MR navigators^9^, ^10^, ^11^, ^12^, ^13^ or head trackers.^14^, ^15^, ^16^, ^17^, ^18^ In this work, we focused on two motion correction techniques based on a rapid 3D fat‐navigator (3D FatNavs)^19^ and on the Tracoline (TCL) markerless motion tracking system developed by TracInnovations.

Three dimensional FatNavs have been demonstrated to enable the detection and correction of non‐deliberate motion for high resolution imaging in compliant subjects, both when implemented as a retrospective technique^19^ and for use with real‐time correction.^20^ The natural sparsity of fat images makes it possible to apply the GRAPPA parallel imaging technique^21^ at exceptionally high acceleration factors, allowing a very rapid acquisition of a high‐resolution navigator that can detect and correct for even very small motion.

The TCL (v3.01) is a 3D structured‐light based stereo vision system that enables head motion tracking for PET, MRI, and simultaneous PET/MRI^22^ without using markers.

In this study, we compared FatNav‐based and TCL‐based motion correction; our aim is to understand which motion leads to the worst artifacts and how well image quality can be restored with the two different motion‐tracking estimates. In the next sections, we will give a short introduction on how retrospective motion correction works and on the FatNav and TCL tracking techniques.

### 1.1 Retrospective motion correction

Retrospective motion correction techniques use Fourier properties to correct the MR k‐space when affected by motion. If bulk motion occurs during the acquisition, the MR signal is affected by a change in phase and magnitude. According to the Fourier shift theorem, translations can be compensated by a phase correction for each point in k‐space along *x*, *y*, and *z* directions, where *x* is left (positive) to right, is up (positive) to down and *z* is head (positive) to foot.^7^, ^23^ On the other hand, rotations, following the Fourier rotation theorem, have the same effect in k‐space and in the image domain. This will lead to effective k‐space sampling that does not fall into a regularly spaced Cartesian grid—also creating regions with lower and higher density points (pie‐slice effect), which can lead to artifacts because of localized Nyquist violations. The non‐uniform fast Fourier transform (NUFFT) can be used to reconstruct this non‐Cartesian sampling and to partially compensate for the artifacts that arise. In our work, we used the NUFFT implementation from Jeffrey Fessler's reconstruction toolbox (https://web.eecs.umich.edu/∼fessler/code/), where Min‐Max interpolation is used to optimally estimate the sampling points, which has been demonstrated to provide lower approximation errors compared to conventional interpolation methods.^24^

### 1.2 Three dimensional FatNavs

The 3D FatNavs consist of applying a 3D gradient echo (GRE) sequence combined with a fat‐selective excitation as a navigator. The acquisition of 3D accelerated FatNav volumes can be incorporated as part of a T_1_‐weighted imaging protocol such as MPRAGE^25^ with only minimal extra scanning time needed (∼2 s for additional GRAPPA calibration for navigators). The 3D FatNav volumes acquired are co‐registered during the post‐processing pipeline using the realign tool in Statistical Parametric Mapping (SPM) (https://www.fil.ion.ucl.ac.uk/spm/).

The use of navigator‐based methods such as 3D FatNavs has the advantage that no extra hardware is required, which makes it more convenient to use than marker‐based tracking methods such as the Moiré phase tracking (MPT) (Metria Innovation), with which it showed comparable results in cases of deliberate and non‐deliberate motion.^26^

### 1.3 TCL

The TCL tracking device uses structured‐light directed toward the subject's face via an infrared‐camera. This produces a series of 3D point‐clouds (∼30 Hz) of the upper right side of the face (typically including one eye, the nose and part of the forehead). The camera operates through an optical fiber bundle attached to an MR compatible mount overlooking the RF head coil acquiring ∼30‐point clouds per second, which are aligned to a reference point cloud created at the beginning of the scan to estimate the motion parameters. The operator fixes the probe to maximize visibility of the subject's face once the participant is positioned on the scanner table and the head coil attached.

Data from the TCL can be used for both prospective and retrospective motion correction, depending on the interface of the system with the scanner and the image reconstruction method.

Frost et al.^27^ successfully tested the device with prospective motion correction between echo‐trains (ETs) (once‐per‐TR) and within ETs in case of stepwise and continuous motion. The system has been recently used for prospective correction of diffusion weighted EPI images,^15^ producing high quality MR images in cases of fast and continuous motion within a 10° range.

### 1.4 Purpose of the study

The aim of this study is to understand how to achieve the best image quality in different motion scenarios, which is clinically relevant to reduce the need for rescans. To allow a direct comparison, the motion correction based on motion estimates from the two tracking techniques was applied retrospectively to the same data.

## 2 METHODS

### 2.1 Image acquisition

Data were collected from a group of nine healthy subjects on a 3 T Prisma scanner (Siemens Healthineers) using a 64 channel RF Coil array for signal reception. All subjects were scanned with an MPRAGE sequence at 1 mm isotropic resolution with TI/TE/TR = 1100/3.03/2410 ms and FA = 8° (orientation sagittal, phase encoding anterior–posterior). Following each readout train of the MPRAGE, a 3D FatNav navigator was acquired at 4 mm isotropic resolution (TE/TR 1.43/3.4 ms, TA = 0.37 s), for a total scanning time of 5:38 min. Autocalibration lines (ACS) for the FatNavs were acquired once at the beginning of the scan to perform GRAPPA reconstruction.

Calibration of the TCL data was performed at the end of the acquisition via the TracSuite software (v3.0.74), which involves aligning the reference point cloud from the TCL system to the surface of a structural MR volume of the whole head. The first MPRAGE scan from each session, without deliberate motion, was used for this calibration procedure.

### 2.2 Ethics board consent

Ethical approval for this study was obtained from Cardiff University School of Psychology Ethics Committee board. Written informed consent was obtained from all subjects before the study.

### 2.3 Motion experiments

Subjects were asked to follow the instructions given on an MR compatible screen positioned inside the scanner and visible via a mirror attached to the head coil. The mirror was positioned so that the participant could clearly see the screen and the TCL camera field of view was not affected. Instructions were coded using PsychoPy v. 30^28^ and consisted of a dot moving in different directions on screen: participants were asked to follow the dot with their nose so that movements could be carried out in a controlled way.

**FIGURE 1:**
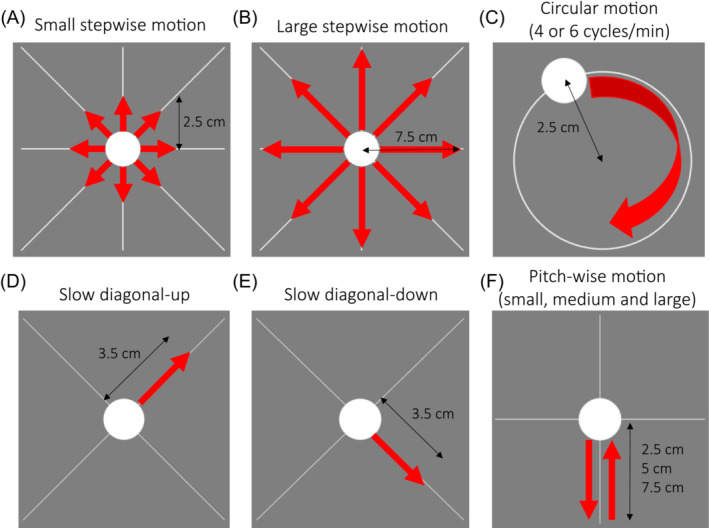
Projected dot motion directions for (A) small stepwise motion (head position changing every 35 s), (B) large stepwise motion (head position changing every 35 s), (C) circular motion at 4 or 6 cycles/min (total head motion of 3 min over 5:38 min of total scan time), (D) slow diagonal motion along the up‐right diagonal (total head motion 1:30/5:38 min), (E) slow diagonal motion along the down‐right diagonal (total head motion 1:30/5:38 min), and (F) pitch‐wise motion (head motion 17 s/min). The dot projected movement is reported (in cm) for each motion scenario. The predicted head motion was estimated based on the eye‐to‐screen distance (76 cm) as: (A) 1.9°, (B) 5.7°, (C) 1.9°, (D,E) 2.6°, and (F) 1.9°, 3.8°, and 5.7° for small, medium, and large pitch‐wise motion, respectively.

Different types of motion were conducted, here, referred to as “stepwise,” “circular,” and “simulated realistic” motion, which are shown in Figure 1. During stepwise motion (Figure 1A,B), the dot moved in a “cross” shape: up, down, right, left, and along two diagonals (up‐right, down‐left; up‐left, down‐right), changing position every 35 s. The projected dot movement was 2.5 cm and 7.5 cm for small and large stepwise motion respectively, with an expected head motion of 1.9° and 5.7° based on the eye‐screen distance of 76 cm.

Circular motion (Figure 1C) was performed similarly to Frost et al.^27^ with the participant's head following a dot moving in circle (radius of 2.5 cm) for 1 min at different speeds: 6 cycles/min and 4 cycles/min. The motion was performed three times during a single MPRAGE acquisition: at the beginning, half‐way through and toward the end of the scan, for a total of 3 min of motion over 5:38 min of scan time. For the dimensions used, the expected maximum head deflection was ∼1.9°.

Finally, simulated realistic motion patterns were generated, based heuristically on an example of existing motion traces in a non‐compliant subject during an fMRI experiment, acquired without deliberate motion, where the subject seemed to move predominantly along the x‐axis performing abrupt pitch rotations or slowly moving their head throughout the acquisition. Therefore, we derived two other types of motion: “slow diagonal” motion (Figure 1D,E) and “pitch‐wise” motion (Figure 1F). The aim was to test the correction methods with what we considered more “realistic” motion. In our pitch‐wise (or nodding) motion scenario, the dot moved quickly down in 2 s, moved up to resting position in 15 s and stayed still for 35 s, for a total motion time of 17 s/min. The projected dot moved vertically on the screen for 2.5 cm, 5 cm, or 7.5 cm, corresponding to small, medium, and large levels of motion, for an expected pitch rotation of 1.9°, 3.8°, and 5.7°. In our slow diagonal motion case, the subject was moving the head slowly for 1:30 min, starting from the center along the up‐right diagonal or the down‐right diagonal (projected motion of dot: 3.5 cm; expected head deflection: 2.6°).

One MPRAGE without deliberate motion was also acquired as a motion‐free reference image for each session.

We obtained a total of 11 datasets, with subjects 3 and 4 being scanned twice on different days. We will refer to different acquisitions of the same experiment as “runs.” Table 1 details the experiments performed by each subject, summarized here as:

- Large and small stepwise, with head motion every 35 s.
- Circular motion at 6 cycles/min and 4 cycles/min, with 3 min of motion per 5:38 min of total scan time.
- Small, medium and large pitch‐wise, with a total motion time of 17 s/min.
- Slow diagonal‐up and slow diagonal‐down, with 1:30 min of motion per 5:38 min of total scan time.

**TABLE 1:** Summary of the experiments performed for each subject.

Experiments performed for each participant	
Subject 1	Still, small pitch‐wise, slow diagonal up, slow diagonal down
Subject 2	Still, large pitch‐wise, medium pitch‐wise, circular‐6 cycles/min
Subject 3	Still, large stepwise, small stepwise, large stepwise, small stepwise
Subject 4	Still, large stepwise, small stepwise, large stepwise, small stepwise, circular‐4 cycles/min
Subject 5	Still, large stepwise, small stepwise, circular‐4 cycles/min
Subject 6	Still, large stepwise, small stepwise, circular‐6 cycles/min
Subject 7	Still, circular‐4 cycles/min, circular‐6 cycles/min
Subject 8	Still, circular‐4 cycles/min, circular‐6 cycles/min
Subject 9	Still, circular‐4 cycles/min, circular‐6 cycles/min

### 2.4 Motion quantification

The motion score is a single value motion metric used by Tisdall et al.^10^ to estimate the motion occurring during each TR. It is defined in Eq. 1 as:

(1) $$\text{score}=∆R+\left({∆}_{2}^{x}+{∆}_{2}^{y}+{∆}_{2}^{z}\right),$$

with ${∆}_{x}^{2},{∆}_{y}^{2},{∆}_{z}^{2}$, being the estimated translations along *x*, *y*, and *z*.

∆R (Eq. 2) is the largest displacement experienced by any point on a sphere of 64 mm radius rotated by an angle θ (Eq. 3).

(2) $$\begin{array}{cc} & ∆R=64\sqrt{(1-\cos(\theta )){\;}^{2}+\sin(\theta ){\;}^{2}} \end{array}$$

(3) $$\begin{array}{cc} \left|\theta \right| & =\left|\arccos\frac{1}{2}\left[-1+\cos\left(\theta_{x}\right)\cos\left(\theta_{y}\right)+\cos\left(\theta_{x}\right)\cos\left(\theta_{z}\right)\right.\right.\\ & \;\left.\left.+\;\cos\left(\theta_{y}\right)\cos\left(\theta_{z}\right)+\sin\left(\theta_{x}\right)\sin\left(\theta_{y}\right)\sin\left(\theta_{z}\right)\right]\right| \end{array}$$

We calculated the mean motion score from each motion estimate and used it as a single value to represent rotational and translational motion over the whole scan. We also estimated the expected motion score based on each type of prescribed motion, with the corresponding expected head motion calculated from the projected dot movement.

### 2.5 Image reconstruction

The image reconstruction was performed MATLAB (The MathWorks) using the retroMoCoBox toolbox.^29^ Because the TCL data displayed high frequency noise in the original motion parameters, a low‐pass filter (lowpass MATLAB function) was used to smooth the motion estimates before the reconstruction using a cutoff frequency of 1 Hz (chosen heuristically based on visual appearance of motion curves). The details regarding the choice of the filter can be found in the Supporting information (Figures S1–S6). All the results reported in this publication were generated after applying this low‐pass filter.

### 2.6 Image quality assessment

The image quality after the motion correction was assessed visually and using two different mathematical metrics: the feature similarity index (FSIM)^30^ as a reference‐based metric and the normalized gradient squared (NGS)^31^ as a non‐reference‐based metric. We considered it important to include both, as a non‐reference‐based metric might be expected to be particularly useful in clinical practice where a good reference image is not always available.

The FSIM was chosen as it has been shown to achieve higher consistency with radiologist evaluations of image quality than other metrics,^32^ including the commonly used structural similarity index.^33^ The primary feature used to calculate the FSIM is the phase congruency, which is a robust spatial frequency‐based system able to identify similarities at the edges: Fourier components (here calculated from a magnitude‐image) with high phase congruency values identify features with sharp changes between light and dark areas, which are what we visually perceive as edges. Because phase congruency is contrast invariant, the gradient magnitude was added as the second factor of this metric. FSIM requires a reference image for comparison and its value varies between 0 to 1, where 1 is obtained when the two images being compared are identical.

The quality of the acquired images was also assessed with the NGS, which has been found by McGee et al.^31^ as the second‐best quality metric for autofocusing, which correlated most closely with observer judgments on MRI images of the shoulder, after the gradient entropy. NGS allows the evaluation of the image quality without comparing it with a reference and postulating that ideal images should have areas of uniform brightness separated by sharp edges. It has been used by Lin et al.^34^ because of its lower computational cost compared with the gradient entropy, and Bazin et al.^35^ chose it as a metric as expected to be more sensitive than the entropy of gradients to limited motion. The NGS has also been successfully used by Gretsch et al.^26^ to compare the quality of images after FatNav and MPT motion correction. NGS values are expected to increase as the image becomes sharper. A mathematical description of the two metrics can be found in the Supporting information.

Before all metrics were calculated, each 2D slice was independently normalized to values from 0 to 1. To estimate the FSIM metric, an extra rescale step between 0 to 255 was required. Final values were estimated averaging them over the 30 central axial slices (consistent with method used by Frost et al.)^27^

To test the improvement quantified by the image quality metrics after the motion correction, a Wilcoxon signed rank test (*signrank* MATLAB function) was performed on motion scenarios with sample size ≥6.

### 2.7 Improving FatNavs motion estimation

When FatNav volumes are acquired, a strong signal can be detected in the neck region as well as around the scalp. The scalp can be expected to move reasonably rigidly with the head (and brain), whereas the neck movement is non‐rigid. The standard processing for 3D FatNavs in the retroMoCoBox software is to use SPM^36^ to perform 6‐degrees of freedom rigid‐body alignment between FatNav volumes to generate motion estimates. If more signal is acquired in the non‐rigid neck region this will affect the quality of the motion estimates, an effect that is particularly noticeable using the Siemens 64‐channel RF coil, because it is a “head and neck” coil with receive channels extending into the neck region. We, therefore, tested whether masking the non‐rigid part of the head would improve the motion parameter estimation and image quality in all our motion scenarios. We expected the mask to be particularly beneficial in the case of strong pitch‐motion, as this direction of motion is likely to have the largest discrepancy between head movement and apparent motion in the neck.

To generate the mask corresponding to the parts of the scalp expected to move rigidly (and therefore, allowing exclusion of non‐rigid regions), we first selected the T_1_‐weighted (T_1_w) image of one dataset acquired without deliberate motion and the corresponding first FatNav volume. We registered the T_1_w and the FatNav volume using the FSL FMRIB's Linear Image Registration Tool function,^37^, ^38^ to have a 3D FatNav and T_1_w image in the same space. After applying brain extraction tool (BET),^39^, ^40^ we registered the T_1_w volume to the 1 mm MNI152 standard space brain.^41^, ^42^ By following the same process, a 3D FatNav for each subject could be brought into a standard space, and then averaged using *fslmaths* from the FSLutils^43^ to obtain a standardized FatNav volume. ITK‐SNAP^44^ was used to manually define a mask in this standard space that would exclude the neck region. When estimating the motion parameters for each subject from the FatNavs, the first FatNav from the subject was co‐registered to the standardized FatNav volume, allowing the mask to be brought into subject‐space and incorporated as a weighting image to SPM's *spm_realign* function. Statistical difference between the image quality obtained by using FatNav‐based motion correction with and without the neck mask was assessed using the Wilcoxon signed rank test (*signrank* MATLAB function).

## 3 RESULTS

3

### 3.1 Comparison between FatNavs and TCL motion correction

**FIGURE 2:**
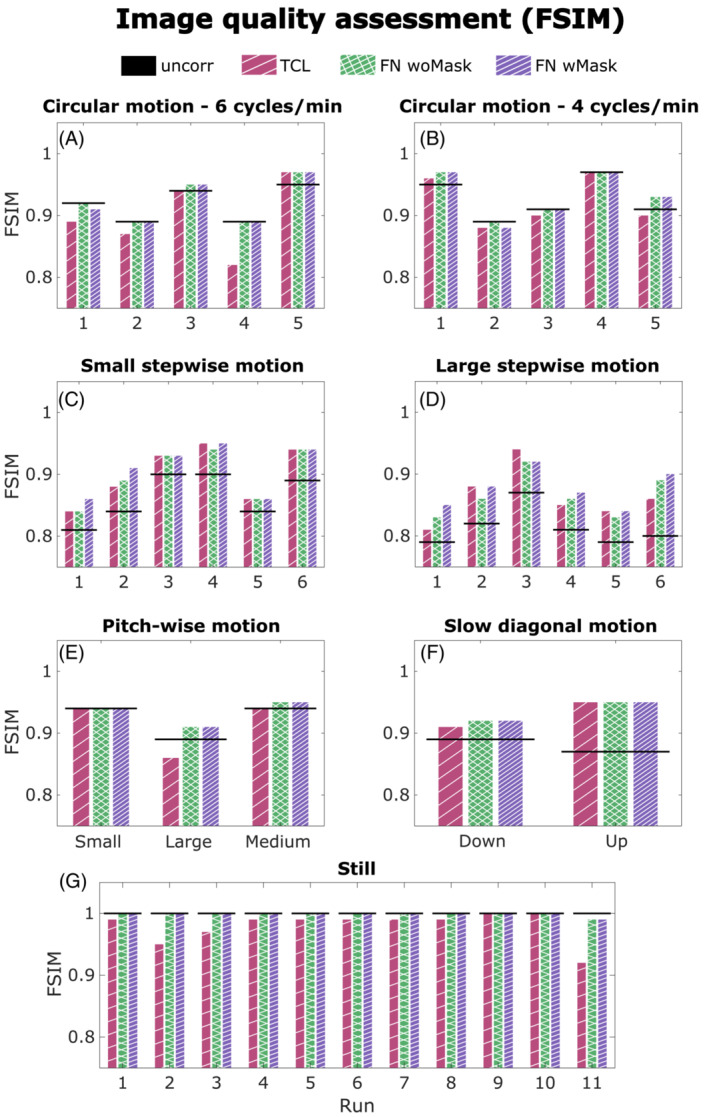
Comparison of feature similarity index (FSIM) values (against the reference image) of TCL‐based corrected (red), fat‐navigator (FatNav) without (“FN woMask” in green) and with mask (“FN wMask” in purple) corrected images obtained from the runs performed for each motion scenario. The FSIM value of the initial uncorrected image is reported as a straight black line.

Figure 2 summarizes all the FSIM values (measured against the “still” image) for all motion scenarios and the three different motion correction methods. The FSIM score is shown to improve by applying all motion correction methods in our small and large stepwise motion scenarios (W = 0, *p* < 0.001), as well as in presence of slow diagonal (up and down) motion (*n* = 2, no statistical test performed). In our small and large stepwise motion scenarios, a substantial improvement in the sharpness was obtained by masking the non‐rigid part of the head for the FatNav co‐registration step (W = 91.5, *p* < 0.001), with only small residual artifacts still visible: the use of the neck‐mask for FatNavs improved the image quality in runs 1, 2, 4, 5, and 6 for large stepwise motion and 1, 2, and 4 of small stepwise motion shown in Figure 2 (“FN wMask”), compared to when no mask is used (“FN woMask”).

**FIGURE 3:**
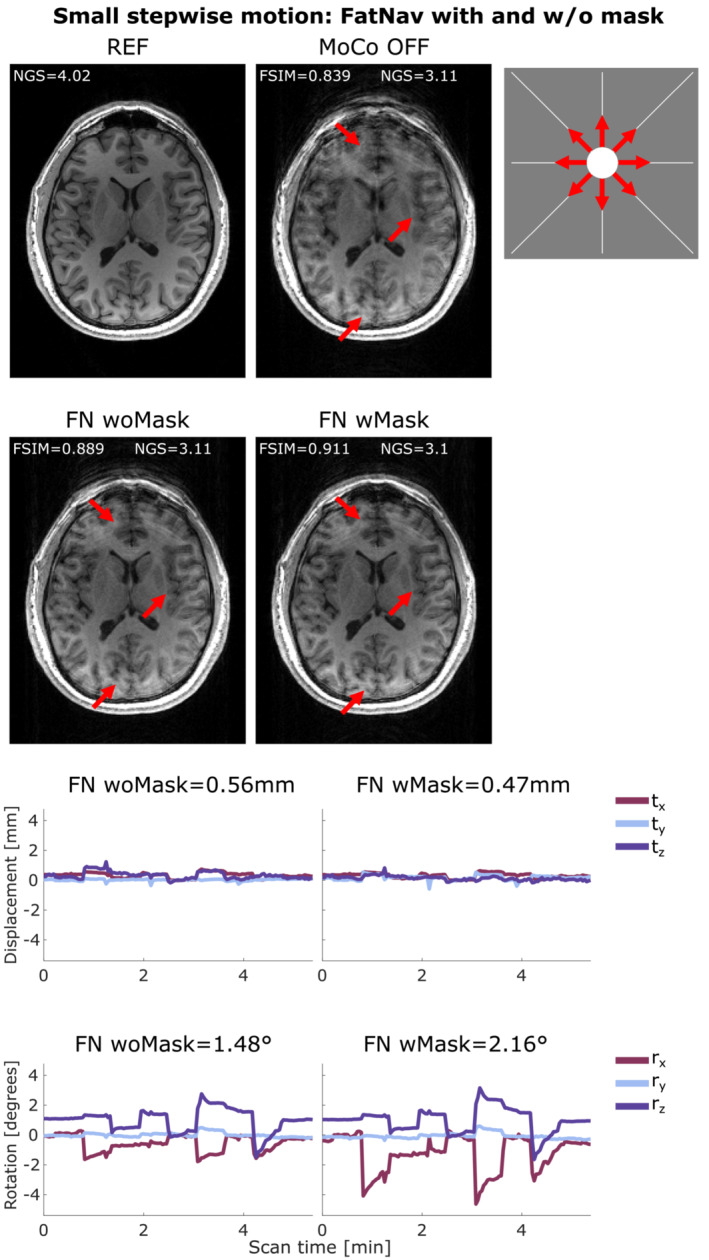
(A) Comparison between reference (REF), uncorrected (MoCo OFF), and the corrected images (FN woMask, FN wMask) for small stepwise motion: the NGS and FSIM values are reported for each image. (B) Motion parameters estimated by the FN woMask (left column) and the FN wMask (right column) tracking methods, with the RMS value reported on top of each motion trace for translations (in mm) and rotations (in degrees). The motion was timed to start 20 s after the beginning of the scan, with the head position changing every 35 s after that. The total scan duration was 5:38 min. The red arrows indicate areas where the FN wMask motion correction improved the image sharpness compared to the uncorrected image and FN woMask. FN wMask, fat‐navigator with mask; FN woMask, fat‐navigator without mask; FSIM, feature similarity index; NGS, normalized gradient squared.

**FIGURE 4:**
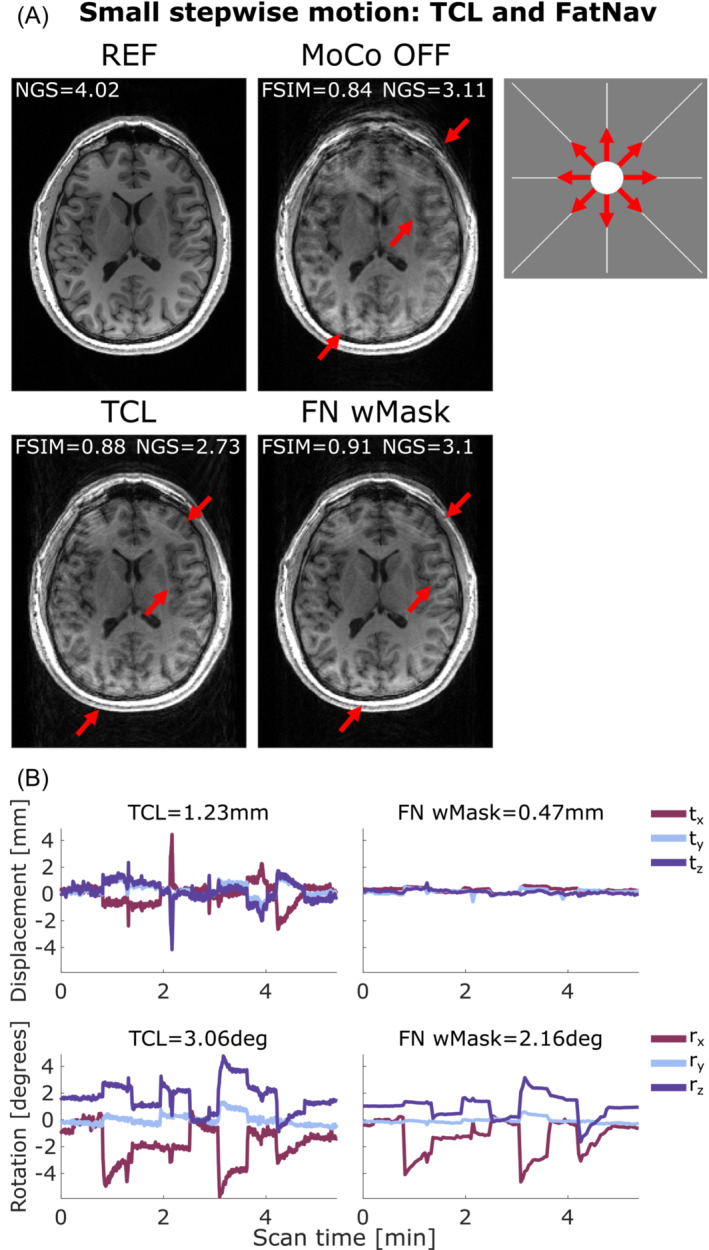
(A) Comparison between reference (REF), uncorrected (MoCo OFF), and the corrected images (TCL, FN wMask) for small stepwise motion: the NGS and FSIM values are reported for each image. (B) Motion parameters estimated by the TCL (left column) and the FN wMask (right column) tracking methods, with the RMS value reported on top of each motion trace for translations (in mm) and rotations (in degrees). The motion was timed to start 20 s after the beginning of the scan, with the head position changing every 35 s after that. The total scan duration was 5:38 min. The red arrows indicate where the motion correction improved the image quality compared to the uncorrected image. Moreover, FN wMask helped reducing the ringing artifacts in the posterior region of the brain further compared to the TCL correction. FN wMask, fat‐navigator with mask; FN woMask, fat‐navigator without mask; FSIM, feature similarity index; NGS, normalized gradient squared; TCL, Tracoline.

Figure 3 compares the motion parameters and MPRAGE images from the FatNav‐based tracking for run 2 of small stepwise motion reported in Figure 2. Removing the neck‐region during the FatNavs registration resulted in a noticeably higher FSIM value (Figure 2) and clear improvements in the image quality, as judged by visual observation (Figure 3). The masked‐FatNav and the TCL corrections for the same experimental run are compared in Figure 4. Here, the top parts of the image (front regions of the brain) were clearly made sharper by the TCL correction. However, the overall best motion correction was obtained using masked‐FatNav estimates. Although some artifacts are still visible toward the front of the brain, this correction noticeably reduced the ringing artifacts in the posterior of the brain compared to the TCL method.

**FIGURE 5:**
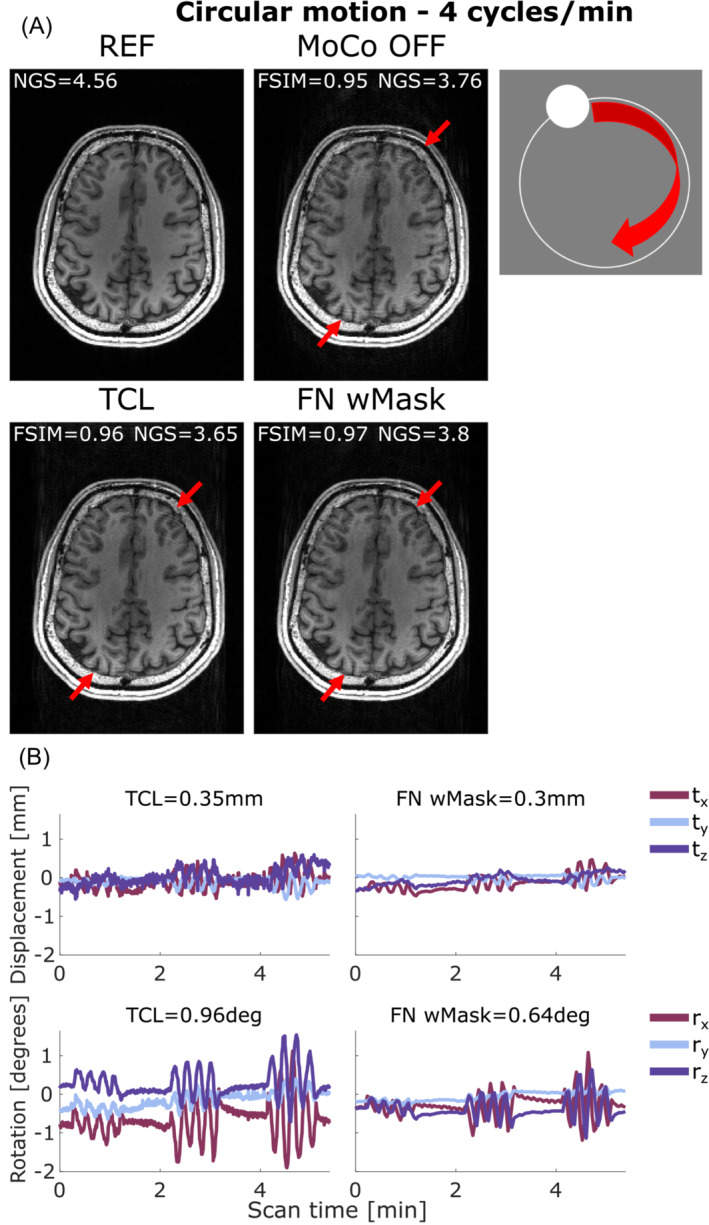
(A) Comparison between reference (REF), uncorrected (MoCo OFF), and the corrected images (TCL, FN wMask) for circular motion at 4 cycles/min: the NGS and FSIM values are reported for each image. (B) Motion parameters estimated by the TCL (left column) and the FN wMask (right column) tracking methods, with the RMS value reported on top of each motion trace for translations (in mm) and rotations (in degrees). The motion was timed to start 10 s after the beginning of the scan, continue for 1 min followed by 1 min without voluntary motion and repeated other two times, for a total head motion time 3/5:38 min. The red arrows indicate areas where the two motion correction methods reduced the ringing artifacts affecting the uncorrected image. FN wMask, fat‐navigator with mask; FN woMask, fat‐navigator without mask; FSIM, feature similarity index; NGS, normalized gradient squared; TCL, Tracoline.

For circular motion at 6 cycles/min and circular motion at 4 cycles/min the outcome is more nuanced, with no clear improvement in image quality metrics following correction (W = 13.5, *p* = 0.32 for FatNav and W = 41, *p* = 0.18 for TCL). There are also some examples of cases where the motion‐correction even appears to lead to an apparent loss of image quality (i.e., a reduction in FSIM following the application of the motion‐correction). Figure 5 illustrates an example of circular motion with the participant performing head rotations at 4 cycles/min (run 1 in Figure 2). The ringing artifacts visible on the uncorrected image were successfully reduced (although not fully eliminated) by correcting using FatNav and TCL motion‐estimation techniques, leading to a better image quality.

For pitch‐wise motion (*n* = 3, no statistical test performed), subjects moved at three different magnitudes for each run, following a projected dot movement of 2.5 cm (1.9°) for run 1, 7.5 cm (5.7°) for run 2, and 5 cm (3.8°) for run 3, corresponding to small, large, and medium pitch‐wise motion (images not shown). The artifacts were almost undetectable in run 1 (2.5 cm), as the subject movement had such low magnitude. In run 2 (7.5 cm), TCL‐based correction led to an apparent degradation of the image quality measured by the FSIM. Despite some small improvements visible toward the front of the brain, the posterior of the brain displayed strong artifacts, probably caused by the abrupt nod motion or facial movements that reduced the tracking accuracy. During run 3 (5 cm), ringing artifacts were reduced by both FatNavs (with and without mask) and TCL, especially toward the front of the brain, improving the image quality compared to the uncorrected image (medium pitch‐wise motion case in Figure 2E).

**FIGURE 6:**
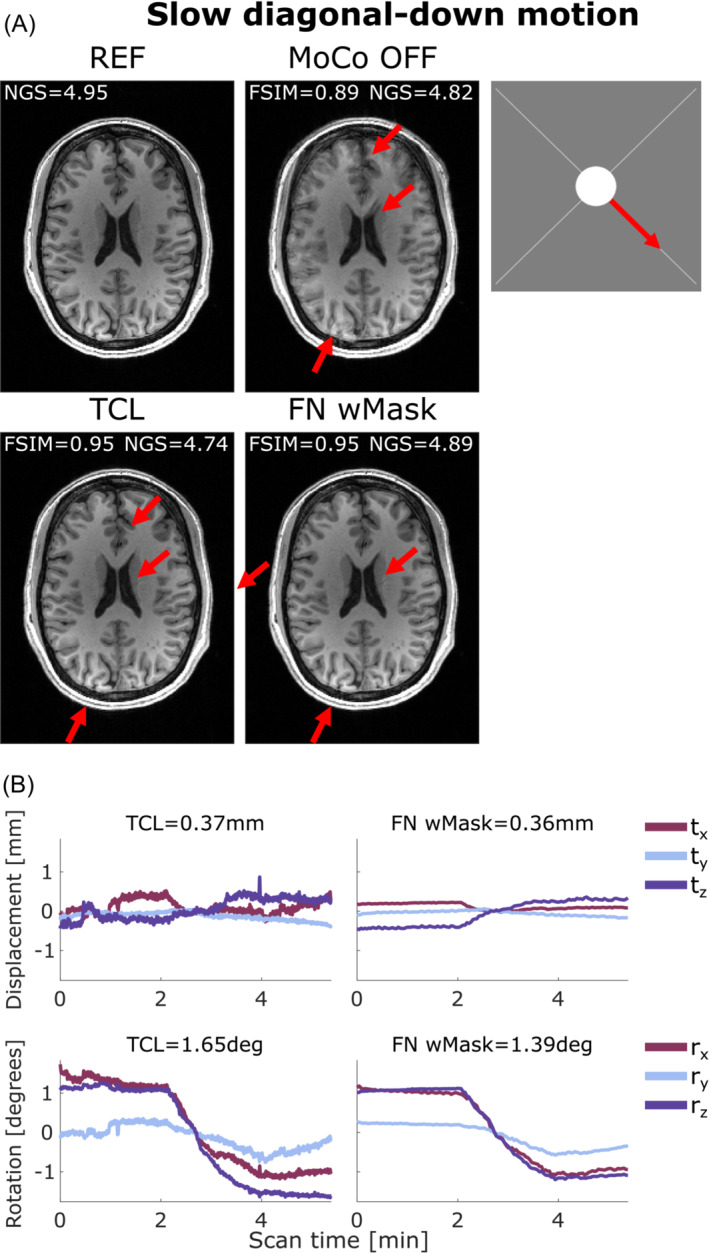
(A) Comparison between reference (REF), uncorrected (MoCo OFF), and the corrected images (TCL, FN wMask) for slow diagonal‐down motion: the NGS and FSIM values are reported for each image. (B) Motion parameters estimated by the TCL (left column) and the FN wMask (right column) tracking methods, with the RMS value reported on top of each motion trace for translations (in mm) and rotations (in degrees). The motion started 2 min after the beginning of the scan and continued for 1:30 min, for a total head motion of 1:30/5:38 min. The image artifacts visible on the uncorrected image were well‐corrected using both motion correction methods as evidenced by the red arrows. FN wMask, fat‐navigator with mask; FN woMask, fat‐navigator without mask; FSIM, feature similarity index; NGS, normalized gradient squared; TCL, Tracoline.

Both FatNavs and TCL performed well when applied to retrospectively correct the images affected by slow motion across the two diagonals. One example is illustrated in Figure 6 where the motion‐corrected images from both FatNavs and TCL motion estimates are sharp and clear, with no visible residual artifacts.

When comparing between motion‐estimation methods, no clearly better correction was found for any of the motion scenarios. However, the FSIM values after FatNav motion correction are shown to be very close to 1 in all runs of our acquisition without deliberate motion (Figure 2G), meaning close‐to‐perfect matching with each corresponding reference image. The good sharpness displayed by the reference images shown in Figure S6 corroborated that FatNav motion correction did not introduce any degradation when no deliberate motion was performed. Moreover, TCL is shown to have a FSIM score comparable with “FatNavs with Mask” in only two runs of the still scenario (namely runs 9 and 10 in Figure 2), suggesting that the image quality decreased because of artifacts originating from noise in the TCL motion estimation, especially in runs 2, 3, and 11.

### 3.2 Motion quantification

**FIGURE 7:**
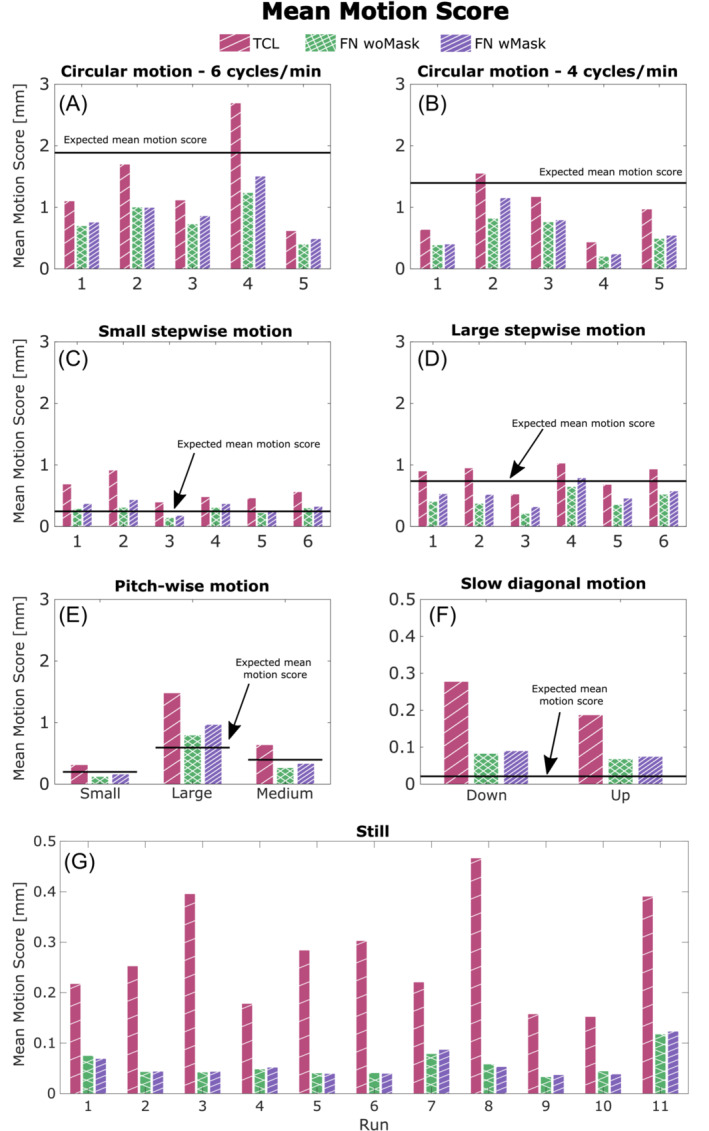
Quantifying the magnitude of the estimated motion: comparison of the motion scores estimated for each run of our motion scenarios. Each motion score was calculated from the motion parameters measured by our three motion tracking modalities: TCL (red), FN woMask (green) and FN wMask (purple). The expected motion score (based on the eye‐to‐screen distance and the projected dot motion) is reported as a black horizontal line. The y‐axis for the still and slow diagonal motion cases (F,G) was ranged differently (from 0 to 0.5 instead of from 0 to 2 mm) to easily see the difference in the motion score values between tracking techniques. FN wMask, fat‐navigator with mask; FN woMask, fat‐navigator without mask; TCL, Tracoline.

**FIGURE 8:**
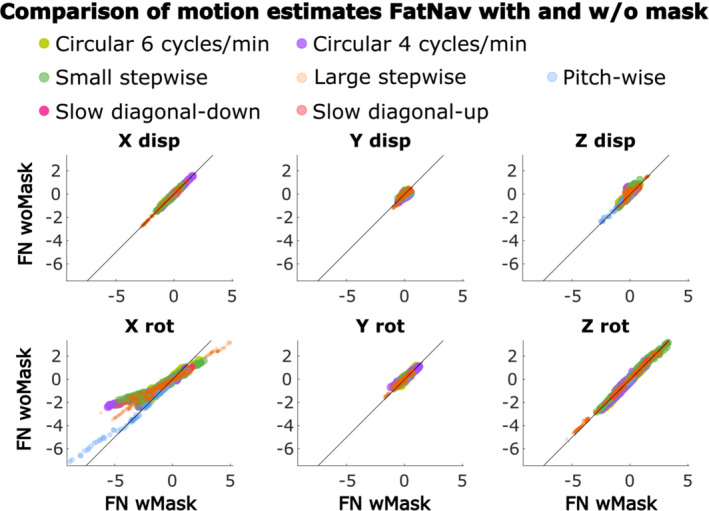
Comparing motion estimates from FN wMask and FN woMask. Each color represents one experiment performed for that type of motion across all subjects: circular motion at 6 cycles/min, circular motion at 4 cycles/min, large stepwise motion, small stepwise motion, pitch‐wise motion (comprehensive of small, medium and large), slow diagonal‐down, slow diagonal‐up. The rotation around the x‐axis (“X rot”) is the only parameter where FN wMask and FN woMask noticeably deviate in their motion estimates, as made clear by the divergence of the plots from the line of identity. FN wMask, fat‐navigator with mask; FN woMask, fat‐navigator without mask.

The mean motion score was chosen as a single valued metric to evaluate the amount of motion performed by each subject in each of our motion scenarios. A summary of all the mean motion scores using motion estimates from TCL and FatNav (with and without mask) is reported in Figure 7. One clear observation from this figure is that the magnitude of the TCL‐based motion parameters is larger than the FatNav‐based estimate in most of our experiments. It also demonstrates how differently the motion parameters are estimated by FatNavs with and without the mask, especially in the case of stepwise motion. The large variation in motion scores between runs obtained for all our motion scenarios demonstrates how different participants varied in the magnitude of motion when performing the same motion type (in the top four graphs in Figure 7, the motion shown on the MR projection screen was the same for all subjects for the same motion type). The motion estimates obtained from FatNavs with and without the mask for all motion types are compared using scatter plots in Figure 8. Most of the dots lie close to the identity line (*y* =*x*), apart from for the rotations around the *x*‐axis. This fits our expectation that the nodding motion of the head leads to the strongest deviations from purely rigid motion within the field‐of‐view of the FatNav.

### 3.3 Image quality assessment

Both FatNavs and TCL motion correction are shown to improve the image quality in the presence of small stepwise, circular, and slow diagonal motion, reported in Figures 4, 5, 6, respectively, with the corresponding FSIM quality metric values, which are reported for each correction method, concurring with this observation by increasing after the motion correction. Conversely, the NGS values found for the same motion cases were found to be smaller for the corrected images compared to the uncorrected cases. This implies a reduction in image quality, which is not what appears to have occurred, based on simple visual inspection of the images in Figures 4, 5, 6. More examples of this behavior are found in Figure S7 for the small stepwise motion scenario.

## 4 DISCUSSION

In this study, two tracking techniques were used to retrospectively compensate for different types of motion: small and large stepwise motion, circular motion at 4 cycles/min and 6 cycles/min, small, medium, and large pitch‐wise motion and slow diagonal motion. Motion estimates from both tracking methods could successfully restore image quality in the case of slow diagonal motion and small and large stepwise motion.

Both tracking methods struggled to allow the restoration of good image quality in the case of circular motion: the FSIM‐based image quality metric even decreased after TCL motion correction in some cases despite the high sampling rate (∼30 Hz) compared to FatNavs (∼0.4 Hz). This might be caused by extensive violations of the Nyquist criterion because of the head rotations involved, which could not be compensated by the single‐step NUFFT‐based retrospective reconstruction. It is possible that iterative methods for applying the motion correction, such as autofocusing algorithms, could complement the motion tracking system and may help to reduce some of these residual artifacts, as suggested by Atkinson et al.^45^ Moreover, prospective motion correction using the estimates from the TCL tracking device has been recently demonstrated to be more robust to motion artifacts compared to retrospective motion correction.^8^ Because of the reduced local effect of Nyquist violation, prospective motion correction could be beneficial in the case of strong head rotations, which were not fully compensated by both FatNavs and TCL retrospective motion correction. Future studies will investigate the sampling rate required to accurately estimate head position changes for different motion scenarios, as our results suggested that none of the investigated motion scenarios fully exploited the fast‐sampling rate allowed by the TCL device.

### 4.1 Improving FatNavs motion estimation

In this study, we also demonstrated that FatNavs estimation accuracy can be improved by masking the non‐rigid part of the neck especially when large pitch‐wise motion is involved (our stepwise motion scenarios). Figure 3 shows how the mask improved the quality of the MPRAGE image especially in anterior regions of the brain.

Looking at Figure 2, we can see that the FSIM measure of image quality obtained after masking was slightly lower than the original FatNavs' correction and the uncorrected image in only two experiments involving circular motion. However, a visual inspection of the two volumes did not detect any visually perceived difference in the image quality. The dissimilarities in the FSIM metric values are attributed to being because of the strong background noise arising after the motion correction (see section 4.3 Background ghosting artifacts). In all other cases, the masked FatNavs provided motion estimates that gave a corrected image at least as good as using the original FatNavs. In Video S1, we show an example of 15 co‐registered FatNav volumes with and without the neck mask, comparing the respective non‐rigid and rigid behavior. The video allows visual confirmation of the expected improvement provided by the mask—with noticeably less apparent residual motion in the aligned volumes.

### 4.2 Motion quantification

The retrospective motion‐correction pipeline needs to make an arbitrary choice of which time point during the acquisition should be considered “zero‐motion”, and to move all other data to fit this coordinate frame. We followed the default behavior of the “retroMoCoBox” in that this is chosen to correspond to the time at which the center of k‐space is acquired as this is expected to correspond approximately to the lowest global offset between the images with and without motion correction (of the same data). Discrepancies in the motion estimates around that time point will generate a visual shift of the motion traces. This effect is noticeable in Figure 5, where TCL estimates look shifted compared to FatNavs. However, this effect is not expected to influence the motion‐correction procedure nor the motion‐score estimation, as the latter is based on frame‐to‐frame motion. The image quality metrics estimated for TCL and FatNavs were found overall to be quite similar, indicating that we cannot easily determine from our data which estimates are a better representation of the “true” motion.

### 4.3 Background ghosting artifacts

In some cases, it was found that the motion‐correction led to visibly more ghosting in the image background than the uncorrected image, as shown in Figure S8. As the motion correction applied is based on estimated motion parameters, which might not fully reflect the real motion occurred, discrepancies can arise in the k‐space, potentially leading to ghosting artifacts. We believe this ghosting may also be affecting the interpretation of the FSIM metric. The TCL correction shown in Figure S8 seemed to lead to a reduction in the image quality based on the FSIM value, despite visible improvements in the image sharpness.

To determine the effect of background ghosting to our image quality metric, we compared the FSIM values of all our images with and without applying a 2D background mask. This mask was based on the convex hull of the mask of a simple threshold value. The convex hull was slightly dilated to be sure to retain the CSF/brain boundary. The mask was then applied to the 30 central slices of each volume over which the FSIM was estimated.

The results obtained from a Wilcoxon signed rank test (MATLAB function *signrank*) did not provide significant evidence of the FSIM values being different when applying a mask to the image background, as shown in Figure S9 (W = 183.5, *p* = 0.71 for FatNav and W = 135, *p* = 0.34 for TCL), concluding that the ghosting in the background did not influence the quality metric chosen.

As extremely strong background noise was limited only to a few cases of circular motion, it is possible that no significant result would emerge from a statistical test. We, therefore, analyzed whether the mask could potentially make a difference in only those cases where the background ghosting was extremely strong. This was performed by first estimating the signal power of the background region, which would be cut off by the head mask, as the ratio between the sum of squares of the background and the overall signal. The estimated background power and the difference between the FSIM values with and without mask were shown to correlate significantly (MATLAB function *corrcoef*, *r* (18) = 0.55, *p* = 0.0066), demonstrating that the stronger the background noise, the more the FSIM metric would increase if a background mask was applied. Figure S10 compares the FSIM values after applying the background mask to the same images previously showed in Figure S8. Both FatNavs and TCL correction resulted in an improved image quality based on the FSIM, which was not detected when the mask was not applied.

### 4.4 Image quality assessment

In this study, we found that the FSIM reference‐based metric could give a good indication of the true image quality—generally also agreeing with subjective visual assessment. In our data, the NGS quality metric showed an unclear behavior in our experiments, with changes in its scores not seeming to correlate with what visually seemed like a good improvement from the uncorrected to the corrected image, as shown for small stepwise, circular and slow diagonal motion cases reported on Figures 4, 5, 6, respectively.

**FIGURE 9:**
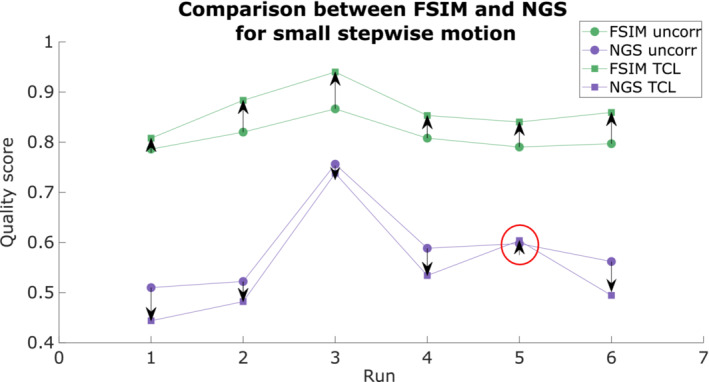
Comparison of feature similarity index (FSIM) and normalized gradient squared (NGS) image quality metrics values of for each run of small stepwise motion estimates from Tracoline (TCL) tracking device. Both metrics were first normalized between 0–1 and the mean value across runs of the experiment subtracted for each metric for display purposes. The circle markers represent the image quality metric value (FSIM in green and NGS in purple) before motion correction; the square markers indicate the metrics' values after the TCL‐based motion correction. Both FSIM and NGS are expected to increase (arrow pointing up) if the image quality improves. The red circle indicates the only experiment in which the FSIM and NGS metrics are in agreement, indicating an improvement in the image quality after motion correction. FatNav, fat‐navigator; FN wMask, fat‐navigator with mask; FN woMask, fat‐navigator without mask.

Figure 9 compares the values from FSIM and NGS for FatNavs with mask and TCL in the case of small stepwise motion. In all our runs, both FatNavs and TCL improved the image quality, as indicated by the FSIM values being higher than for the uncorrected images for all tracking techniques. This was further corroborated by a visual check of all images, which displayed a qualitatively higher level of sharpness compared to when no motion correction was applied (Figure S7). On the other hand, the NGS value altered in the opposite way to what would be expected in all but one run. Further studies will need to be performed to assess the correlation between metrics used to estimate brain MR images quality and radiologist evaluations, which is still to be considered the standard reference. Moreover, additional research is needed to evaluate how these metrics are affected by different artifacts: our results suggest that metrics such as the NGS may not be optimal metrics for driving automated motion‐correction techniques, as we have several examples of a visually “better” image that scores “worse” when judged by NGS.

## 5 CONCLUSIONS

In conclusion, both FatNav‐based and TCL‐based motion‐correction can achieve good image quality in the case of stepwise motion and of slow changes in the head position (our “slow diagonal motion” experiment). When using FatNavs, it is beneficial to also incorporate a mask to exclude non‐rigid parts of the neck to improve the image registration step—this is especially noticeable when larger motion occurs in the pitch‐wise direction as this emphasizes the non‐rigid movement. In the more extreme motion scenarios, the retrospectively corrected images often contained noticeable residual artifacts, which we attribute to violations of the assumptions required for the retrospective correction used. Future work may investigate theoretical limits that will lead to an artifact‐free image after motion correction, elucidating to what extent residual artifacts can be alleviated by more advanced reconstruction techniques or whether real‐time correction may be required when problematic motion scenarios are expected.

In this study, we showed that the use of a reference‐based metric, such as the FSIM, gives a more reliable assessment of the image quality before and after motion correction compared to the non‐reference‐based metrics used. Future studies will focus on testing if this is caused by their different sensitivity to the different manifestations of motion‐related artifacts and how these image quality metrics correlates with neuroradiologists' scores.

## CONFLICT OF INTEREST STATEMENT

Elisa Marchetto received research funding's partially from TracInnovations. Stefan Glimberg is an employee of TracInnovations.

## Supporting information


**Figure S1.** Comparison between the original and filtered data using a low‐pass filter 5 Hz cutoff frequency and a 1 Hz cutoff frequency (30 Hz sampling rate) on 15 s of motion parameters acquired using the TCL device while no voluntary motion was performed (run 11 in Figure 2G in the main document). The motion parameters are displayed in the TCL coordinate system and not in the scanner frame of reference.
**Figure S2.** Comparison between the original and filtered data using a low‐pass filter at 5 Hz and 1 Hz cutoff frequencies (30 Hz sampling rate) on 15 s of motion parameters acquired using the TCL device while no voluntary motion was performed (run 3 in Figure 2G in the main document). The motion parameters are displayed in the TCL coordinate system and not in the scanner frame of reference.
**Figure S3.** Comparison between the original and filtered data using a low‐pass filter at 1 Hz cutoff frequency. The original data were taken from Slipsager et al.^22^ and available here: https://figshare.com/articles/dataset/Tracking_data_Patient_b_/6989336. The figure shows only 1 min of motion parameters for display purposes. The motion parameters are here displayed in the TCL coordinate system and not in the scanner frame of reference.
**Figure S4.** Comparison of the FSIM quality score, calculated against the reference images, in all our motion scenarios with and without using a smoothing function (pink and green, respectively) on the TCL motion parameters before motion‐correction. Based on the FSIM, the smoothing function did not cause any degradation compared to the non‐smooth case, improving or keeping invariant the image quality in our motion scenarios. However, the FSIM score still resulted below the target value of one in our non‐deliberate motion case (still scenario), which was attributed to small tracking biases rather than the noise on the motion traces, because even the smoothed TCL estimates demonstrate a much higher motion score compared to FatNavs for 8 of the 11 “still” runs (all runs except 1, 7, and 10—see Figure 7G in the main document).
**Figure S5.** The effect of smoothing on the TCL‐based motion estimation. Comparison between TCL‐based motion estimation before and after applying the smoothing function: (A) TCL after smoothing (TCL smooth) shows slightly less ringing artifact compared to the unsmoothed version (TCL), which is corroborated by the improvement in the FSIM value. (B) The unfiltered parameters (on the left) are affected by noise, which is partially removed after filtering (right side). The motion parameters are here displayed in the scanner frame of reference.
**Figure S6.** Comparison of all reference volumes acquired without deliberate motion at the beginning of each scan session. No motion correction was applied. All subjects were instructed to stay as still as possible during the scan, which resulted in no visible motion artifacts in the volumes acquired.
**Figure S7.** Uncorrected (MoCo OFF) and corrected images using TCL or FN wMask against the reference image (REF) for all runs of our small stepwise motion scenario. Image quality metrics are reported on each image for comparison between our reference‐based metric (FSIM) and our non‐reference‐based metric (NGS): NGS values imply a reduction in image quality following motion‐correction, despite improvements that are clearly visible compared to the uncorrected image.
**Figure S8.** (A) Comparison between reference (REF), uncorrected (MoCo OFF), and the corrected images (TCL and FN wMask) for circular motion at 4 cycles/min: the corrected images are affected by strong background ghosting, which is not present in the uncorrected image. Images in this figure have been windowed to allow easier visualization of the ghosting rather than optimal viewing of gray/white contrast across the brain. (B) Motion parameters are reported for TCL and FN wMask, with the RMS value reported on top of each motion trace for translations (in mm) and rotations (in degrees).
**Figure S9.** Comparison between the FSIM values of the images without masking the background (“Without mask”) and masking the image background (“With mask”) for FN woMask, FN wMask and TCL.
**Figure S10.** Comparison between the FSIM values of images affected by circular motion at 4 cycles/min (Figure S8) without (“w/o background mask”) and with masking the image background (“with background mask”) in case of TCL, FN woMask and FN wMask motion correction and without motion correction (MoCo OFF).

**Video S1.** Co‐registered sequence of FatNav volumes acquired while the subject was performing circular motion showing the different behavior in the neck area when (A) no mask is applied during the registration process, against (B) when the mask is applied. (B) The created mask is represented in light gray: the neck area is excluded so that only the rigid part of the head is considered during the SPM registration process. The mask is automatically applied to each subject by co‐registering the first FatNav volume to a reference FatNav image in standard space using SPM.

## Data Availability

The data and scripts that support the findings of this study are available from the corresponding author upon reasonable request.
