# Supplementary material for: Robust retrospective motion correction of head motion using navigator‐based and markerless motion tracking techniques
Source: Magn Reson Med. 2023 May 15;90(4):1297–315. doi: 10.1002/mrm.29705 (PMC7615144; doi:10.1002/mrm.29705)
Supplement: Supplementary file 1 — Figure S1. Comparison between the original and filtered data using a low‐pass filter 5 Hz cutoff frequency and a 1 Hz cutoff frequency (30 Hz sampling rate) on 15 s of motion parameters acquired using the TCL device while no voluntary motion was performed (run 11 in Figure 2G in the main document). The motion parameters are displayed in the TCL coordinate system and not in the scanner frame of reference. Figure S2. Comparison between the original and filtered data using a low‐pass filter at 5 Hz and 1 Hz cutoff frequencies (30 Hz sampling rate) on 15 s of motion parameters acquired using the TCL device while no voluntary motion was performed (run 3 in Figure 2G in the main document). The motion parameters are displayed in the TCL coordinate system and not in the scanner frame of reference. Figure S3. Comparison between the original and filtered data using a low‐pass filter at 1 Hz cutoff frequency. The original data were taken from Slipsager et al. 22 and available here: https://figshare.com/articles/dataset/Tracking_data_Patient_b_/6989336. The figure shows only 1 min of motion parameters for display purposes. The motion parameters are here displayed in the TCL coordinate system and not in the scanner frame of reference. Figure S4. Comparison of the FSIM quality score, calculated against the reference images, in all our motion scenarios with and without using a smoothing function (pink and green, respectively) on the TCL motion parameters before motion‐correction. Based on the FSIM, the smoothing function did not cause any degradation compared to the non‐smooth case, improving or keeping invariant the image quality in our motion scenarios. However, the FSIM score still resulted below the target value of one in our non‐deliberate motion case (still scenario), which was attributed to small tracking biases rather than the noise on the motion traces, because even the smoothed TCL estimates demonstrate a much higher motion score compared to FatNavs for 8 of the 11 “stil [file MRM-90-1297-s002.docx]

Robust retrospective motion correction of head motion using navigator-based and markerless motion tracking techniques.

# Supplementary material: Lowpass filter

The raw TCL motion estimates are sampled at ~30Hz (exact data-rate is not constant) and, as a result, exhibit high-frequency fluctuations which are not captured by the slower sampling rate (and slower sampling “shutter”) of the FatNavs. Some of these high frequency fluctuations will be respiratory and cardiac-related motion, and some of it will be due to noise in the noise estimates themselves. To reduce the presence of the high-frequency fluctuations, as we hypothesised that some residual image artifacts could be due to tracking noise, the motion parameters were smoothed using a low-pass filter (*lowpass* MATLAB function using default ‘FIR’ filter). The choice of the cut-off frequency was performed heuristically based on visual appearance of the motion curves as described below.

The filter was tested on a segment of motion parameters acquired on one subject during an acquisition without voluntary motion (run 11 in Figure 2G shown in the main document). Figure S1 compares the TCL motion parameters before and after smoothing using a low-pass filter at 5 Hz or at 1 Hz cut-off frequency. The 1 Hz cut-off frequency is shown to provide a better smoothing compared to the 5 Hz, removing the high-frequency fluctuations.

The effect of smoothing the high-frequency fluctuations using the 1 Hz filter is shown also in Figure S2 against the original unfiltered parameter for a different acquisition without deliberate motion (run 3 in Figure 2G shown in the main document).

We also tested how well this low-pass filter removes the respiratory motion reported in Slipsager et al. 2019 (<https://journals.plos.org/plosone/article/file?id=10.1371/journal.pone.0215524&type=printable>). The data were taken from the website cited in the paper under “Data Availability Statement”. The data used can be found here: <https://figshare.com/articles/dataset/Tracking_data_Patient_b_/6989336>. Figure S3 compares a segment of the original motion parameters and after smoothing using the low-pass filter, displaying a consistent reduction in the tracking noise after filtering.

The low-pass filter was implemented in the retroMoCoBox reconstruction pipeline and applied to all the TCL data collected. Despite reducing the effective sampling rate from 30 Hz to around 2 Hz, the motion parameters smoothed using the low-pass filter did not reduce the image quality in any of our motion scenarios, as shown in Figure S4, overall improving the image quality in some of our motion scenarios (e.g. runs 1, 2, 3, 4 and 6 of small stepwise motion in Figure S4-C), compared to the non-smoothed images, based on the value measure by the FSIM quality metric. One example for large stepwise motion (run 6 in Figure S4-D) is reported in Figure S5. However, the FSIM scores for TCL in our data without deliberate motion (‘still’ scenario), where we would expect a value very close to 1 as we know the motion is very small, remain noticeably lower than 1 in many of the runs, as shown in Figure S4-G. This contrasts with the FatNav correction applied to the same ‘no-motion’ data, which results in an FSIM very close to 1 for all runs except run 11. From the Figure 7 reported in the main document, this difference corresponds to where TCL motion estimates are larger than FatNav-based estimates for the same scans, but as we have no ‘gold standard’ independent measure of the true motion that occurred we cannot immediately say which is more correct. Visual inspection of the corrected vs non-corrected images (Figure S6), however, suggests strongly that the main contribution to this discrepancy is small tracking biases on the TCL estimates.


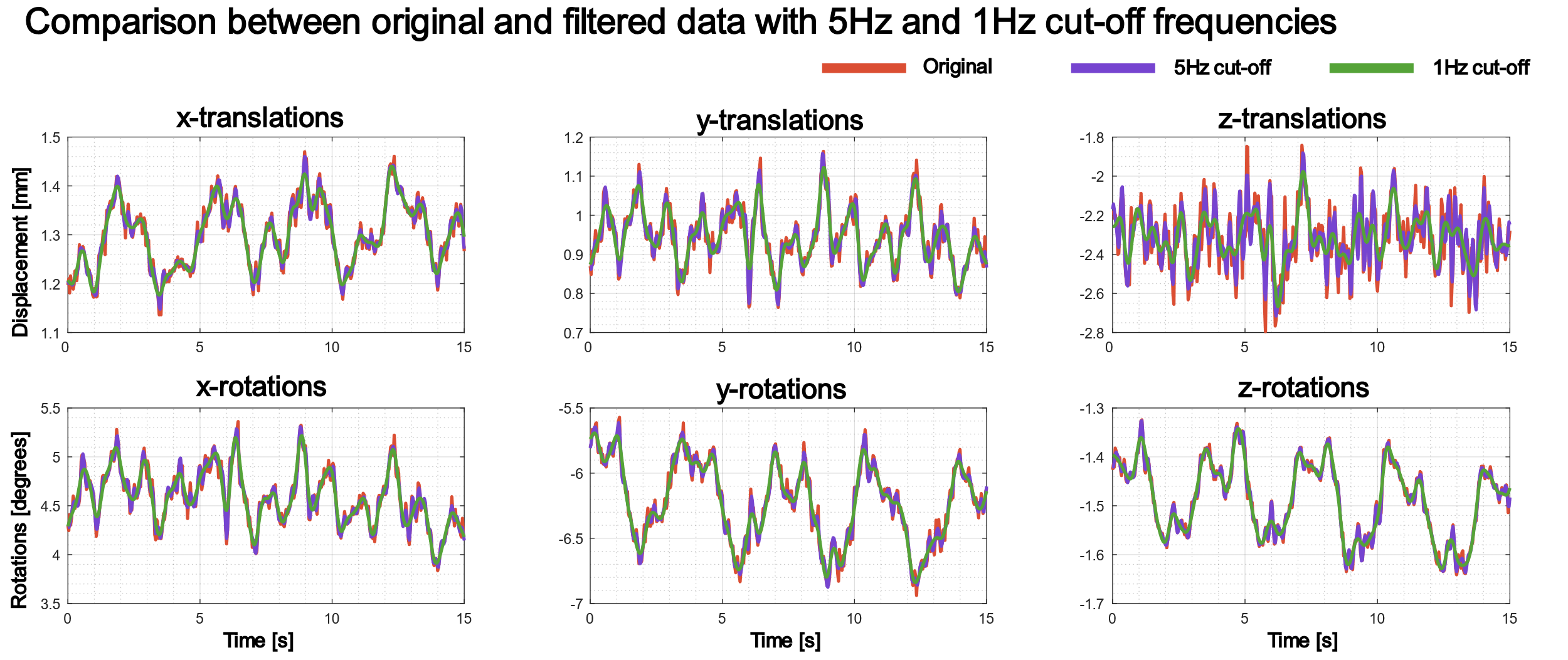


**Figure S1.** Comparison between the original and filtered data using a low-pass filter 5 Hz cut-off frequency and a 1 Hz cut-off frequency (30 Hz sampling rate) on 15 s of motion parameters acquired using the TCL device while no voluntary motion was performed (run 11 in Figure 2G in the main document). The motion parameters are here displayed in the TCL coordinate system and not in the scanner frame of reference.


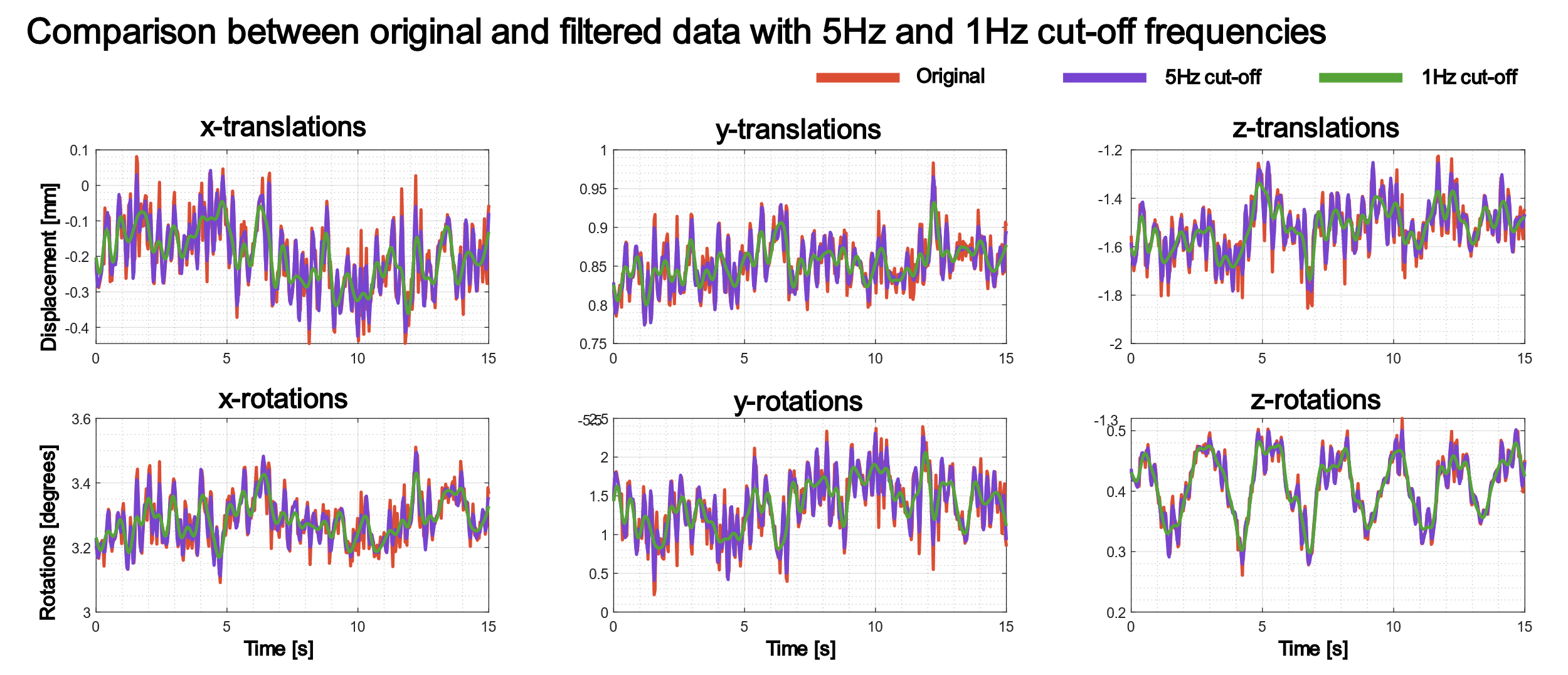


**Figure S2.** Comparison between the original and filtered data using a low-pass filter at 5Hz and 1 Hz cut-off frequencies (30 Hz sampling rate) on 15 s of motion parameters acquired using the TCL device while no voluntary motion was performed (run 3 in Figure 2G in the main document). The motion parameters are here displayed in the TCL coordinate system and not in the scanner frame of reference.


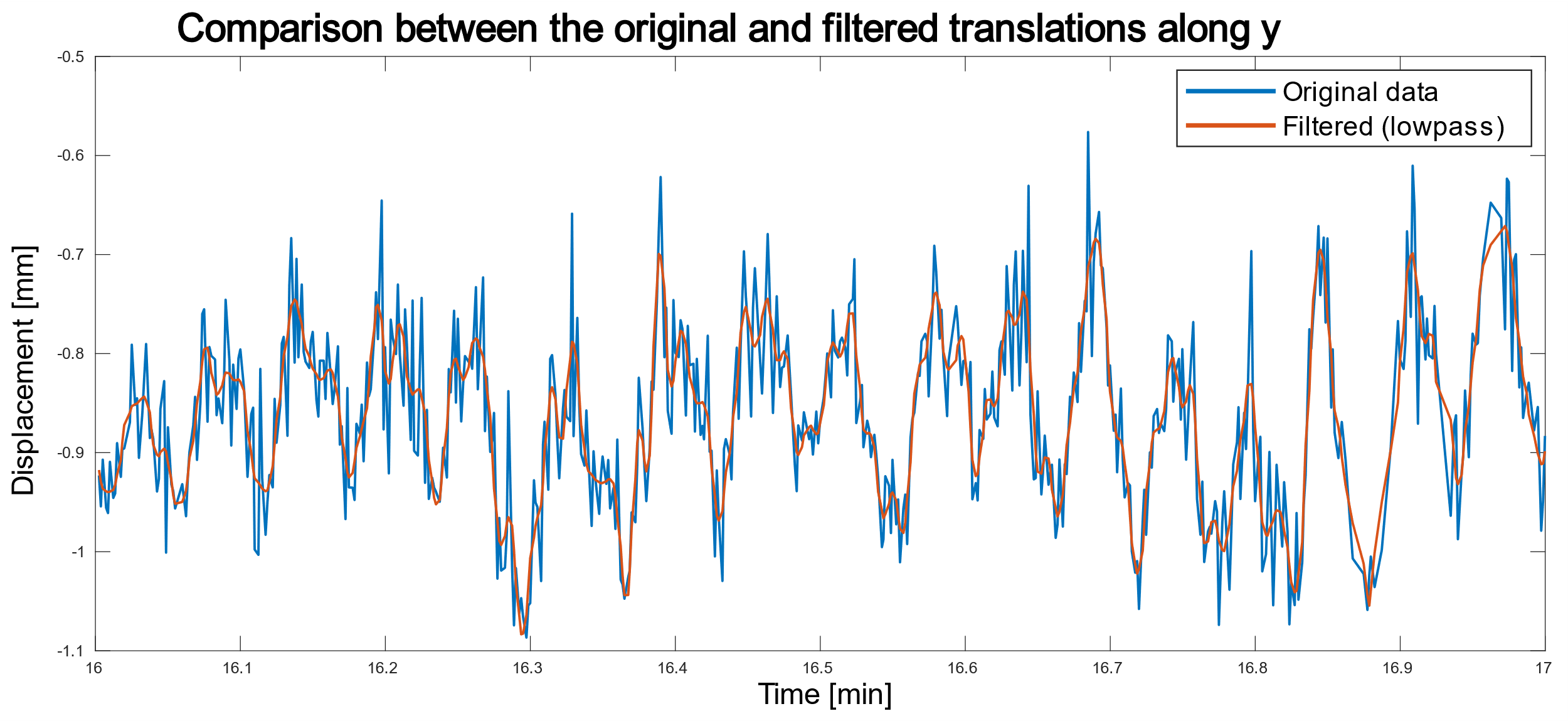


**Figure S3.** Comparison between the original and filtered data using a low-pass filter at 1 Hz cut-off frequency. The original data were taken from Slipsager at al. 2019 and available here: <https://figshare.com/articles/dataset/Tracking_data_Patient_b_/6989336>. The figure shows only 1 minute of motion parameters for display purposes. The motion parameters are here displayed in the TCL coordinate system and not in the scanner frame of reference.


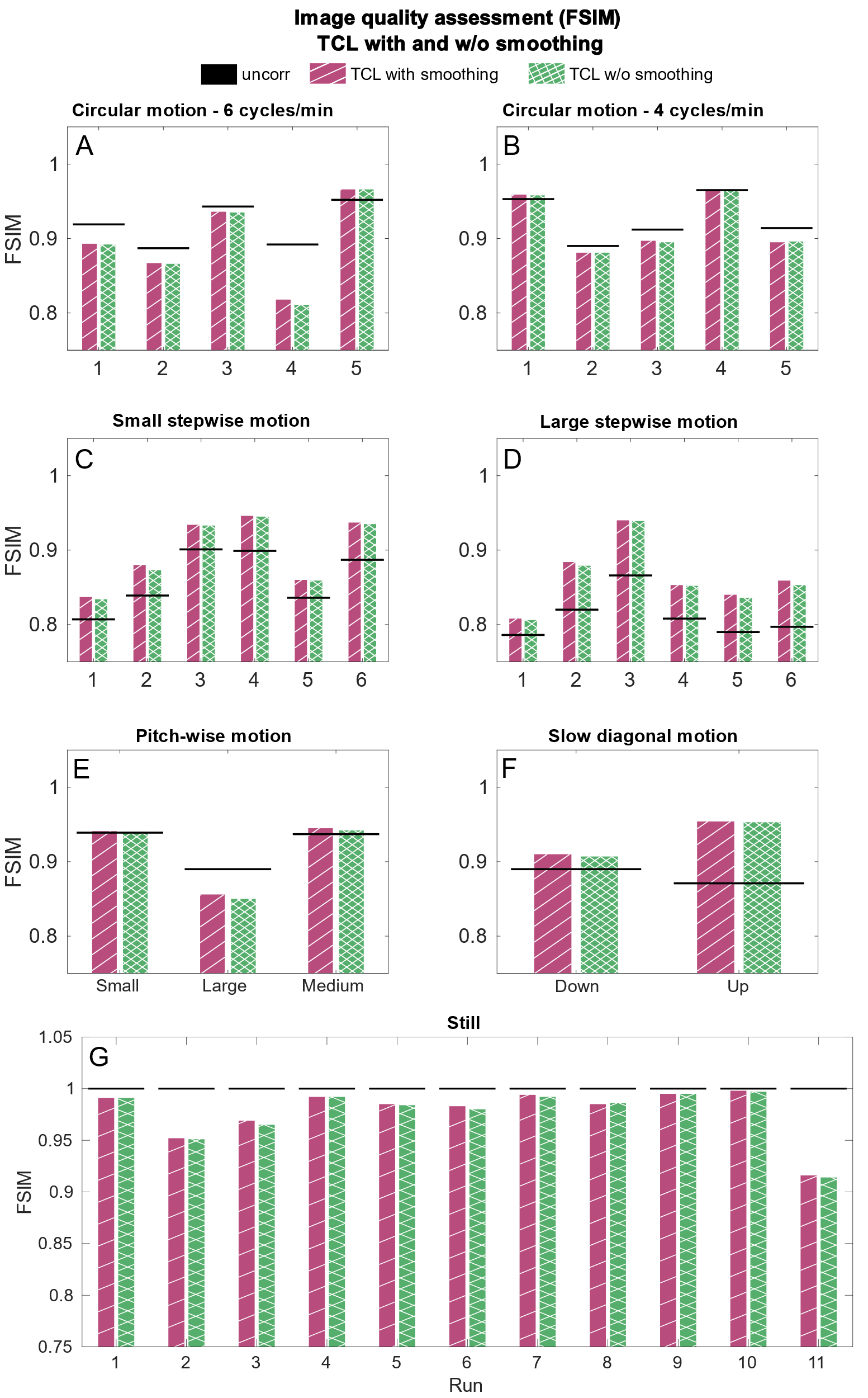


**Figure S4.** Comparison of the FSIM quality score, calculated against the reference images, in all our motion scenarios with and without using a smoothing function (pink and green respectively) on the TCL motion parameters prior to motion-correction. Based on the FSIM, the smoothing function did not cause any degradation compared to the non-smooth case, improving or keeping invariant the image quality in our motion scenarios. However, the FSIM score still resulted below the target value of 1 in our non-deliberate motion case (still scenario), which was attributed to small tracking biases rather than the noise on the motion traces, because even the smoothed TCL estimates demonstrate a much higher motion score compared to FatNavs for 8 out of the 11 ‘still’ runs (all runs except 1,7, and 10 - see Figure 7G in the main document).


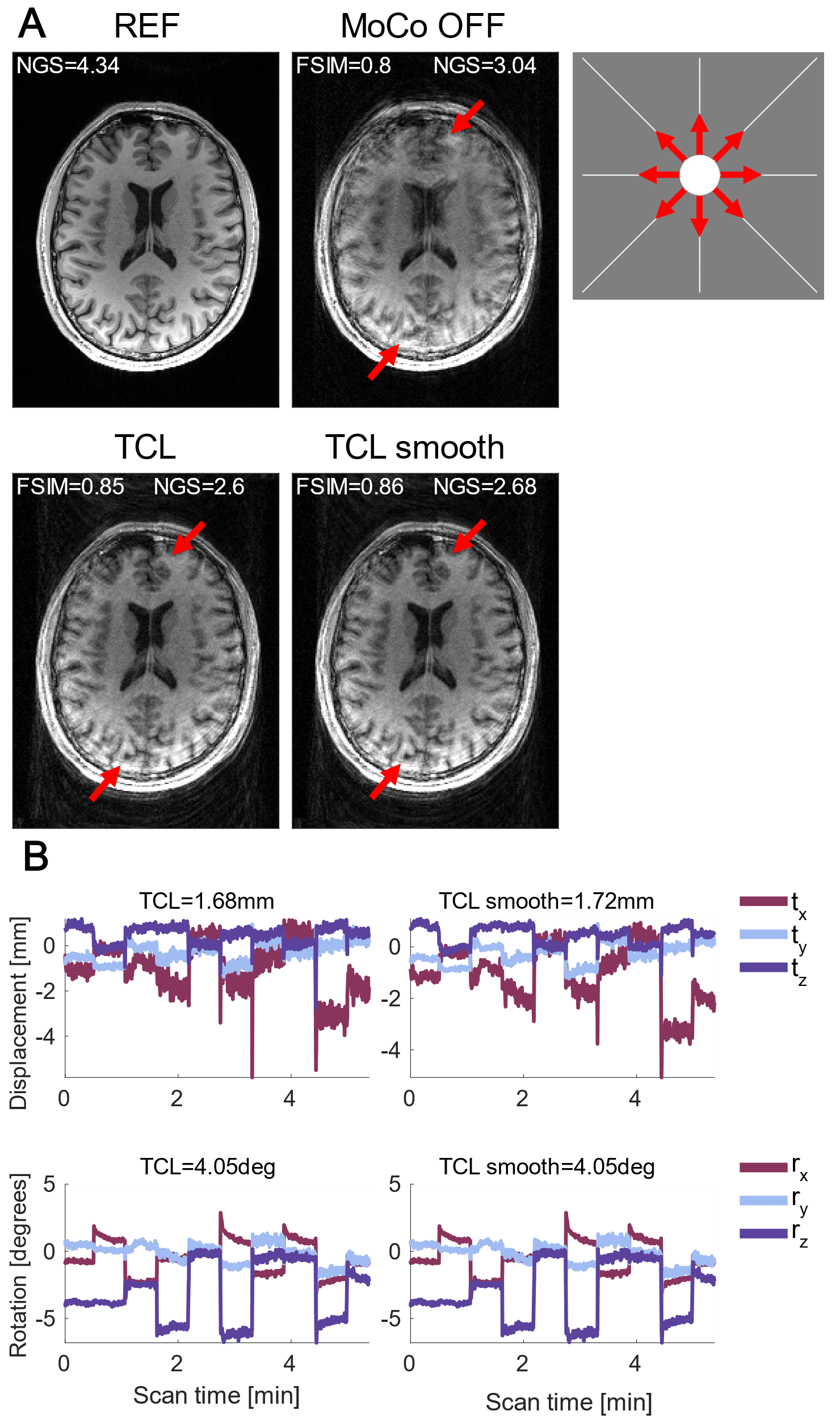


**Figure S5.** The effect of smoothing on the TCL-based motion estimation. Comparison between TCL-based motion estimation before and after applying the smoothing function: (A) TCL after smoothing (TCL smooth) shows slightly less ringing artifact compared to the unsmoothed version (TCL), which is corroborated by the improvement in the FSIM value. (B) The unfiltered parameters (on the left) are affected by noise, which is partially removed after filtering (right side). The motion parameters are here displayed in the scanner frame of reference.


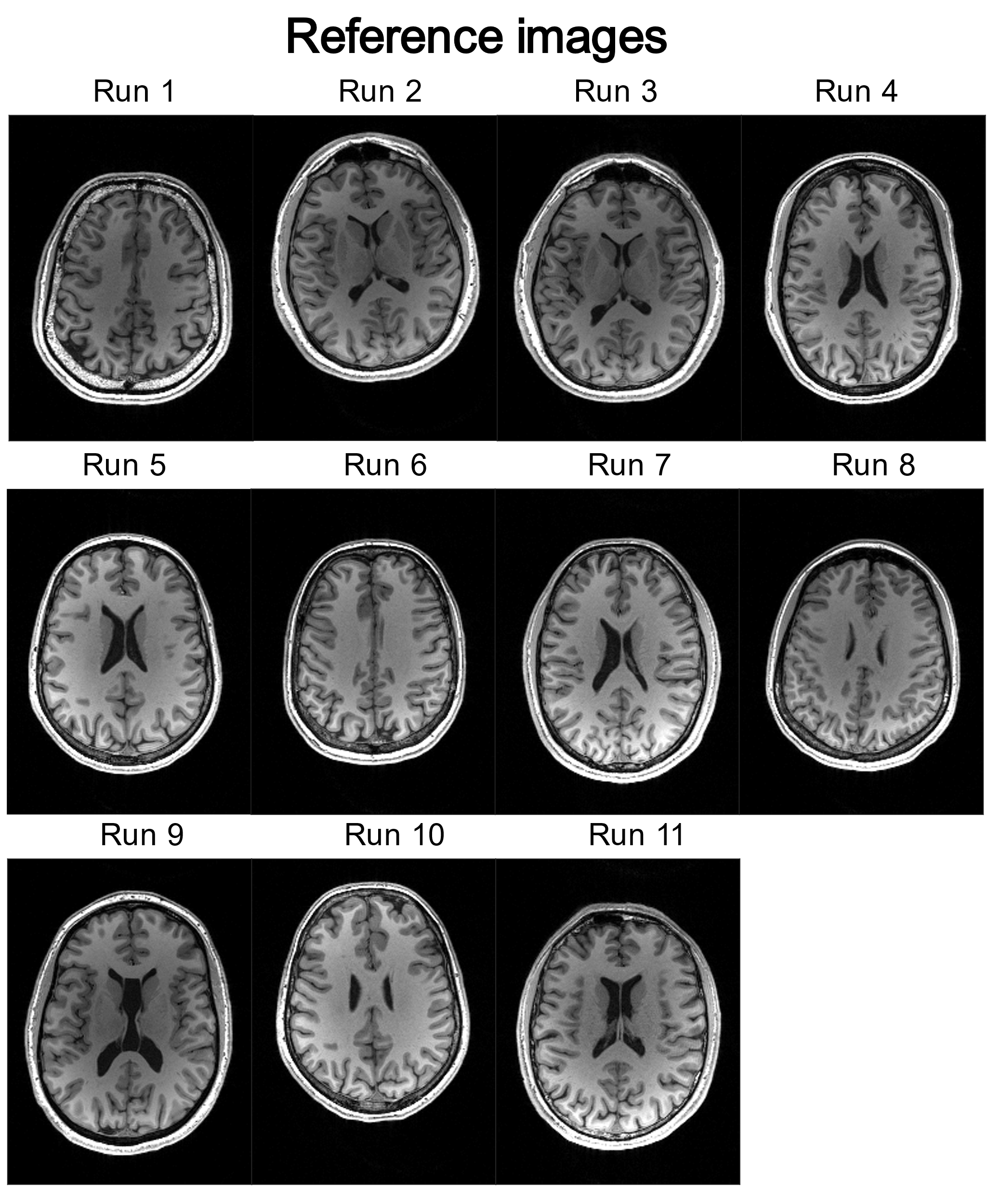


**Figure S6.** Comparison of all reference volumes acquired without deliberate motion at the beginning of each scan session. No motion correction was applied. All subjects were instructed to stay as still as possible during the scan, which resulted in no visible motion artifacts in the volumes acquired.

# Supplementary material – Image quality metrics

In our work, we used two different metrics to evaluate the quality of our brain images: the Feature Similarity Index (FSIM) as a reference-based metric (Zhang et al. 2011) and the Normalized Gradient Squared (NGS) as a non-reference-based metric (McGee et al. 2000). In this supplementary material file, we briefly describe the two metrics mathematically.

## FSIM

The primary feature used to calculate the FSIM is the Phase Congruency (PC), which is a robust spatial frequency-based system able to identify similarities at the edges: Fourier components (here calculated from a magnitude-image) with high PC values identify features with sharp changes between light and dark areas, which are what we visually perceive as edges. The PC for each point $x$ of a 2D image can be mathematically described as follows:

$$\mathrm{PC}\left( x \right)=\frac{\sum_{j} E_{\theta_{j}}\left( x \right)}{\epsilon+\sum_{n} \sum_{j} A_{n,\theta_{j}}\left( x \right)}$$

$E_{\theta_{j}}\left( x \right)$with being the local energy along the orientation j (with j being the orientation angle), and $A_{n,\theta_{j}}\left( x \right)$being the local amplitude on a scale n, and $\epsilon$ a small positive constant. Because the PC is contrast-invariant, the gradient magnitude was added as the second factor of this metric, defined as:

$$G=\sqrt{G_{x}^{2}+G_{y}^{2}}$$

with x and y the two directions along which the gradient was calculated.

The FSIM requires a reference image to be computed and its value varies between 0-1, where 1 is obtained when the two images being compared are identical. Firstly, the similarity map between the image being evaluated and a reference is calculated for the PC and the gradient separately:

$$S_{\mathrm{PC}}=\frac{2PC_{1}\left( x \right)\cdot PC_{2}\left( x \right)+K_{1}}{PC_{1}^{2}\left( x \right)\cdot PC_{2}^{2}\left( x \right)+K_{1}}$$

$$S_{G}=\frac{2G_{1}\left( x \right)\cdot G_{2}\left( x \right)+K_{2}}{G_{1}^{2}\left( x \right)\cdot G_{2}^{2}\left( x \right)+K_{2}}$$

with K_1_ = 0.85 and K_2_ = 160 as in [87]. The two similarities are then combined as:

$$S_{L}\left( x \right)=S_{\mathrm{PC}}\left( x \right)\cdot S_{G}\left( x \right)$$

As areas with high PC indicates sharp edges, the importance of $S_{L}$ is weighted using:

$$PC_{m}\left( x \right)=max\left( PC_{1}\left( x \right),PC_{2}\left( x \right) \right)$$

Finally, the FSIM can be mathematically described as:

$$FSIM=\frac{\sum_{x\epsilon\Omega} S_{L}\left( x \right)\cdot PC_{m}\left( x \right)}{\sum_{x\epsilon\Omega} PC_{m}\left( x \right)}$$

## NGS

The NGS allows the evaluation of the image quality without comparing it with a reference and postulating that ideal images should have areas of uniform brightness separated by sharp edges. The NGS is defined as the normalized convolution between the Prewitt operator and the pixel values $g_{i,j}$:

$$NGS=\sum_{\mathrm{ij}} \frac{\left| \binom{1}{-1}*g_{i,j} \right|}{\sum_{i,j} \left| \binom{1}{-1}*g_{i,j} \right|}$$

with i,j locating each pixel in the image domain.

**
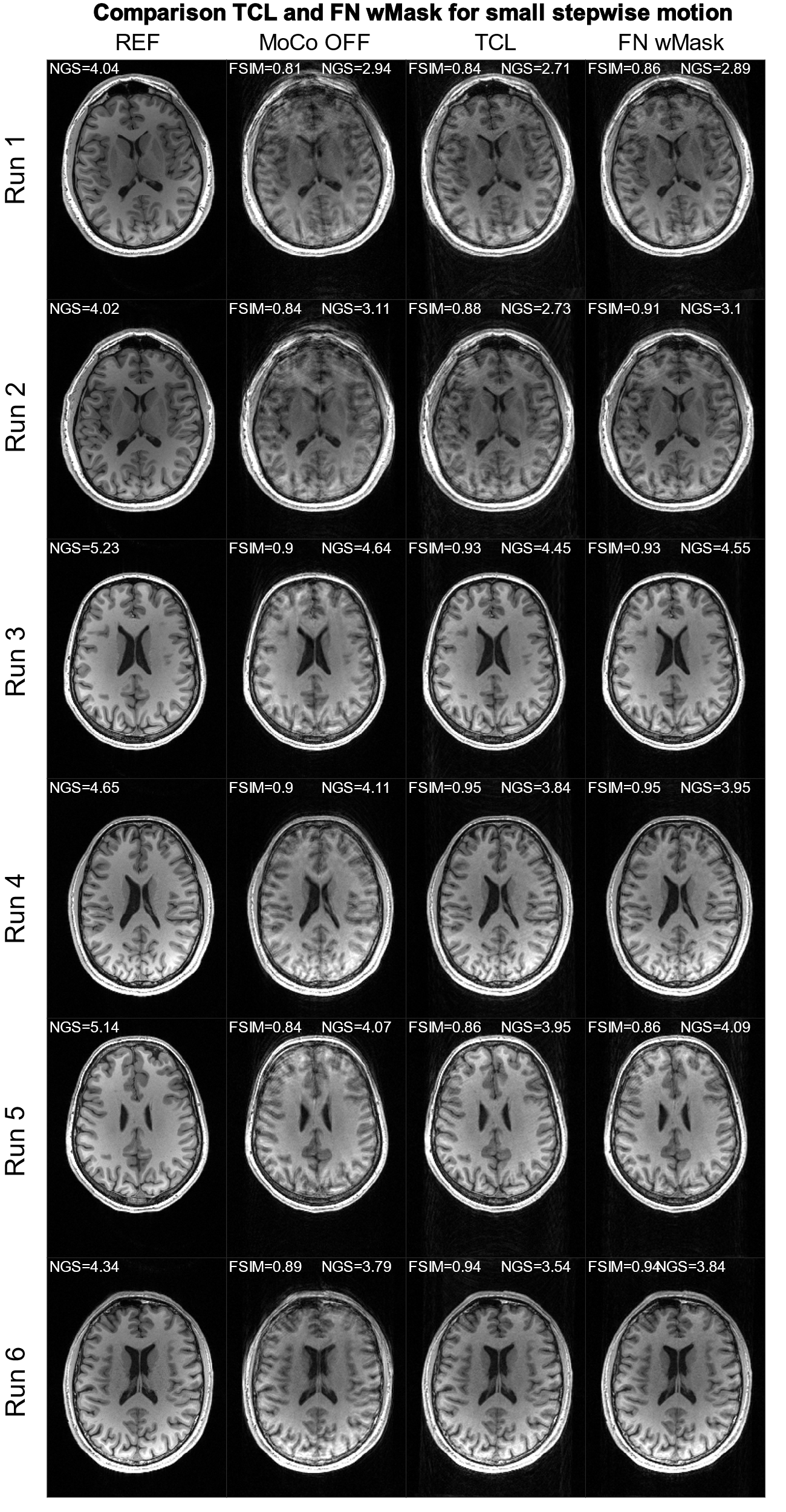
**

**Figure S7.** Uncorrected (MoCo OFF) and corrected images using TCL or FN wMask against the reference image (REF) for all runs of our small stepwise motion scenario. Image quality metrics are reported on each image for comparison between our reference-based metric (FSIM) and our non-reference-based metric (NGS): NGS values imply a reduction in image quality following motion-correction, despite improvements that are clearly visible compared to the uncorrected image.

**
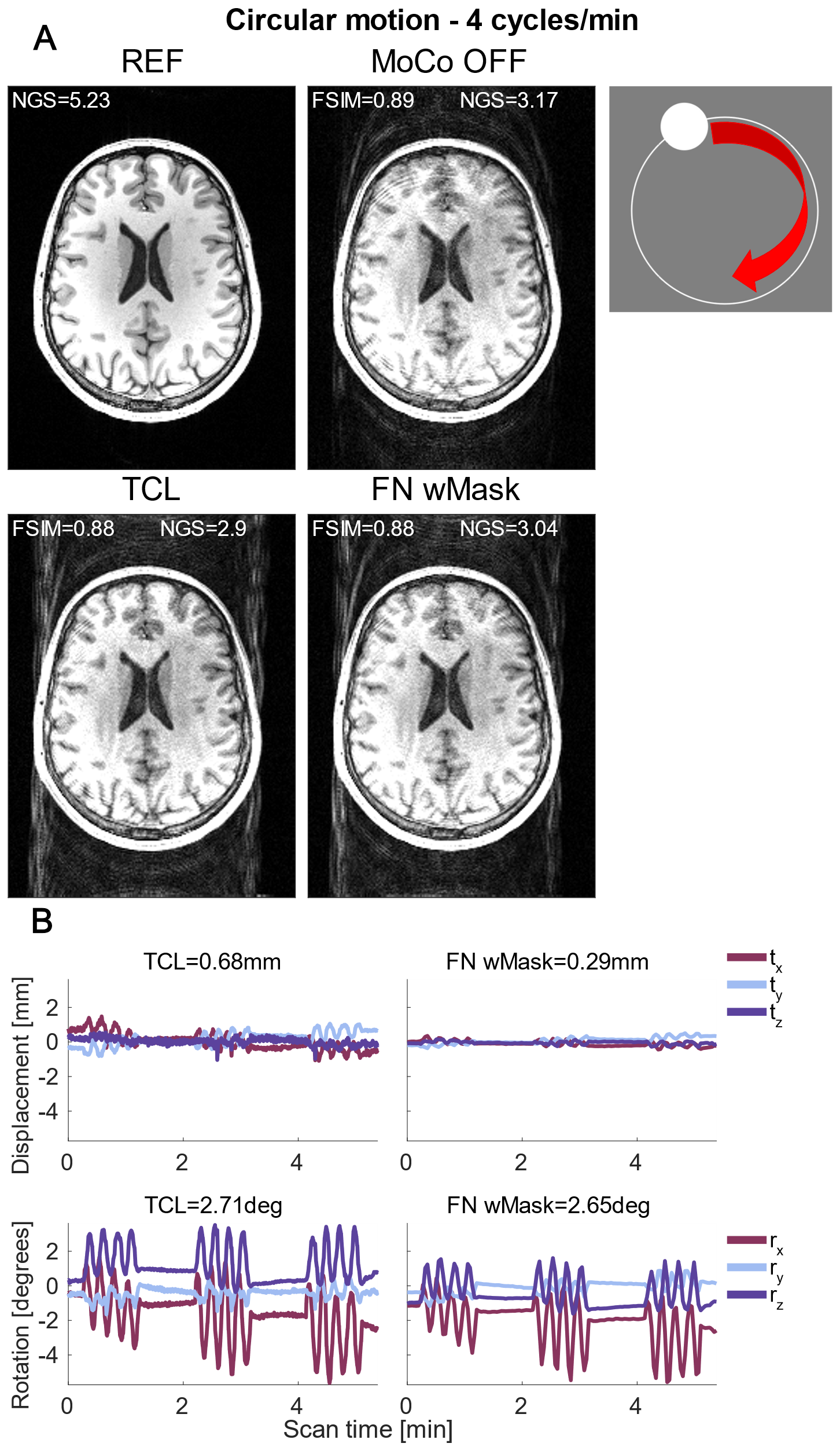
**

**Figure S8.** (A) Comparison between reference (REF), uncorrected (MoCo OFF), and the corrected images (TCL and FN wMask) for circular motion at 4 cycles/min: the corrected images are affected by strong background ghosting which is not present in the uncorrected image. Images in this figure have been windowed to allow easier visualisation of the ghosting rather than optimal viewing of grey/white contrast across the brain. (B) Motion parameters are reported for TCL and FN wMask, with the RMS value reported on top of each motion trace for translations (in mm) and rotations (in degrees).

**
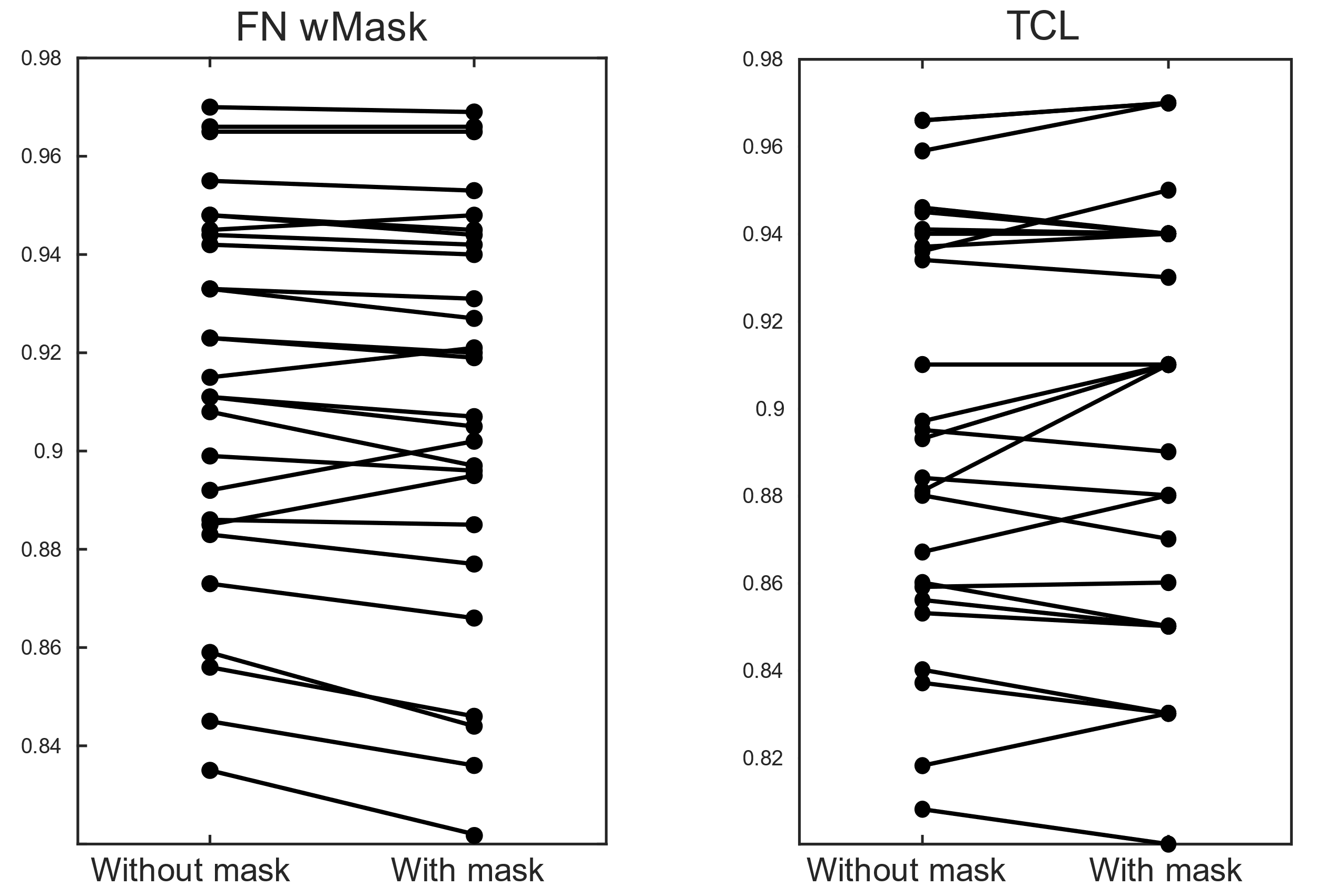
**

**Figure S9.** Comparison between the FSIM values of the images without masking the background (“Without mask”) and masking the image background (“With mask”) for FN woMask, FN wMask and TCL.


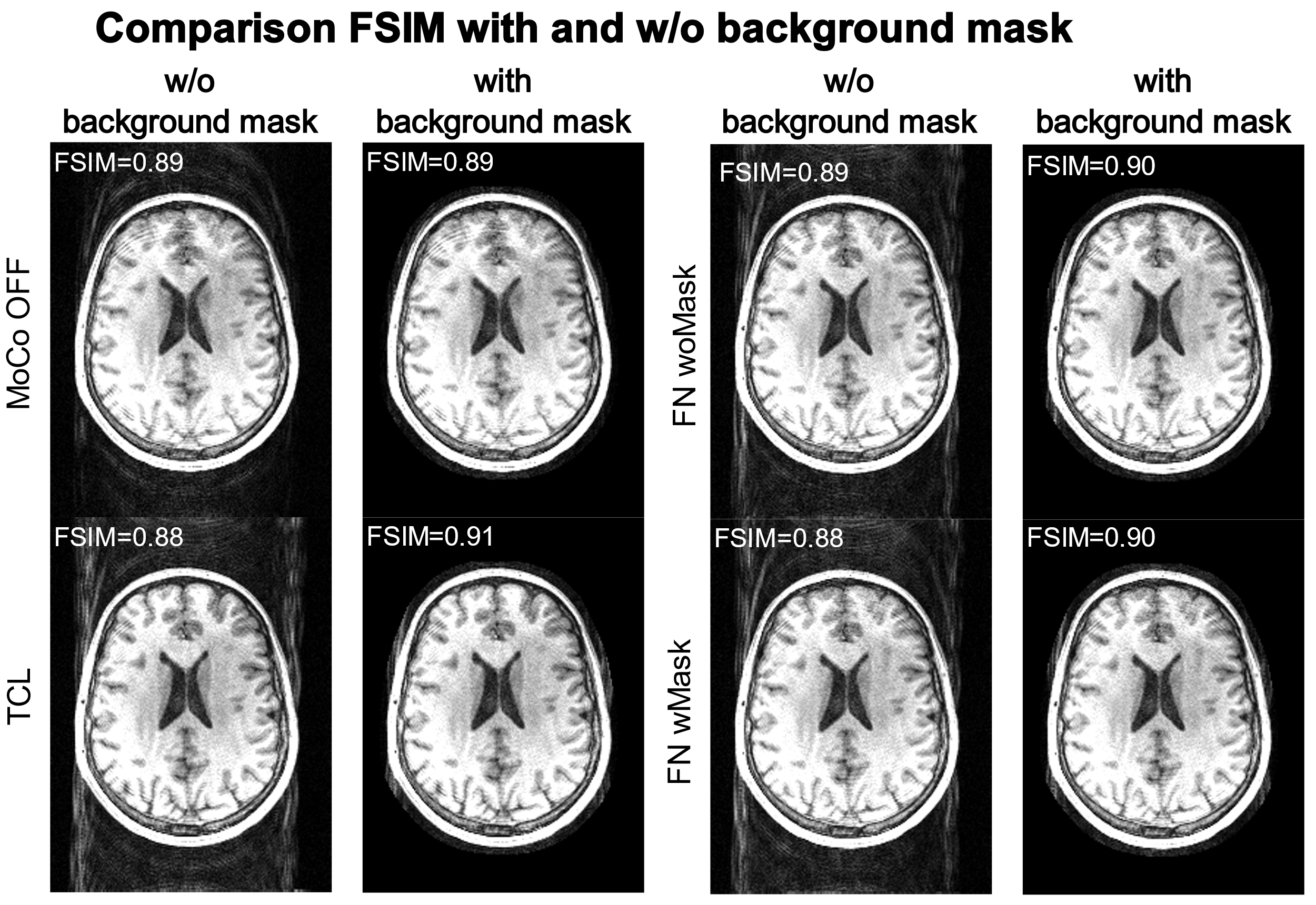


**Figure S10.** Comparison between the FSIM values of images affected by circular motion at 4 cycles/min (Figure S3) without (“w/o background mask”) and with masking the image background (“with background mask”) in case of TCL, FN woMask and FN wMask motion correction and without motion correction (MoCo OFF).
