# Supplementary material for: Robust retrospective motion correction of head motion using navigator-based and markerless motion tracking techniques
Source: Magn Reson Med. Author manuscript; Available in PMC 2023 Oct 2. (PMC7615144; doi:10.1002/mrm.29705)
Supplement: Supplementary file 1 [file EMS188114-supplement-Video_S1.pdf]

with masking the image background ("with background mask") in case of TCL, FN woMask and FN wMask motion correction and without motion correction (MoCo OFF).

Video S1. Co-registered sequence of FatNav volumes acquired while the subject was performing circular motion showing the different behavior in the neck area when (A) no mask is applied during the registration process, against (B) when the mask is applied. (B) The created mask is represented in light gray: the neck area is excluded so that only the rigid part of the head is considered during the SPM registration process. The mask is automatically applied to each subject by co-registering the first FatNav volume to a reference FatNav image in standard space using SPM.

How to cite this article: Marchetto E, Murphy K, Glimberg SL, Gallichan D. Robust retrospective motion correction of head motion using navigator-based and markerless motion tracking techniques. *Magn Reson Med*. 2023;90:1297-1315. doi: 10.1002/mrm.29705

15222594, 2023, 4, Downloaded from https://onlinelibrary.wiley.com/doi/10.1002/mrm.29705 by Test, Wiley Online Library on [18/09/2023]. See the Terms and Conditions (https://onlinelibrary.wiley.com/terms-and-conditions) on Wiley Online Library for rules of use; OA articles are governed by the applicable Creative Commons License
